# Amphiphilic Janus Particles for Aerobic Alcohol Oxidation in Oil Foams

**DOI:** 10.1021/acscatal.4c00909

**Published:** 2024-07-19

**Authors:** Kang Wang, Josh Davies-Jones, Arthur Graf, Marina Carravetta, Philip R. Davies, Marc Pera-Titus

**Affiliations:** † Cardiff Catalysis Institute, School of Chemistry, Cardiff University, Main Building, Park Place, Cardiff CF10 3AT, U.K.; ‡ School of Chemistry, University of Southampton, Highfield, Southampton SO17 1BJ, U.K.

**Keywords:** Janus particles, gas−liquid−solid interface, alcohol oxidation, oil foams, microreactor, photoinduced force microscopy (PiFM)

## Abstract

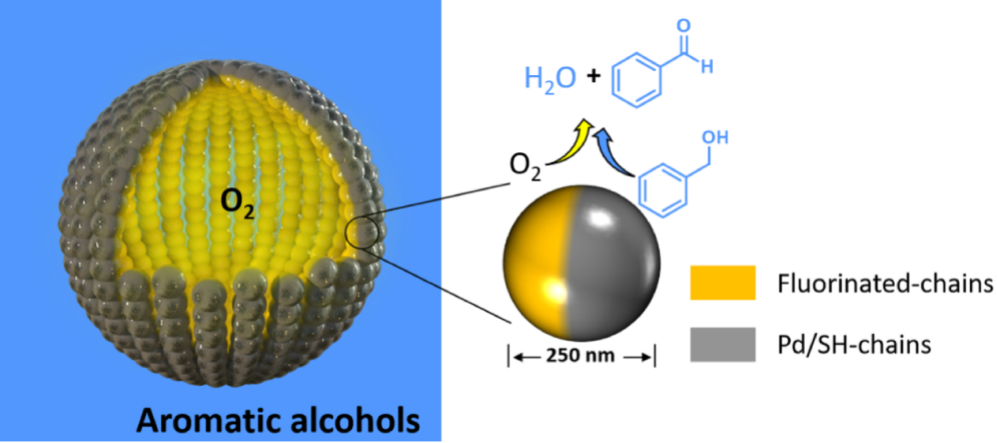

Amphiphilic Janus silica particles, tunable with oleophobic–oleophilic properties and low fluorine content (8 wt % F), exhibited prominent foamability for a variety of aromatic alcohols at low particle concentrations (<1 wt %) compared to randomly functionalized silica particles. When selectively loaded with Pd nanoparticles on the oleophilic hemisphere, the particles displayed more than a 2-fold increase in catalytic activity for the aerobic oxidation of benzyl alcohol compared to nonfoam bulk catalysis under ambient O_2_ pressure. The particles were conveniently recycled with high foamability and catalytic activity maintained for at least five consecutive runs.

## 1 Introduction

Liquid foams are omnipresent in daily life and are widely used in the formulation of food and beverages, cosmetics, healthcare and homecare products, as well as in fire extinguishing, froth flotation, and the manufacturing of porous materials.^1−4^ Foams are typically stabilized using surfactants, surface-active polymers, or globular proteins.^5−8^ These stabilizers exhibit efficacy primarily in aqueous environments and are not readily applicable to organic solvents owing to their low surface tension (typically ranging from 14 to 50 mN·m^–1^). The generation of nonaqueous foams requires stabilizers with low surface energy (such as, fluorinated surfactants, asphaltenes, or fatty acid crystals).^9−12^ This task is considerably more challenging compared to aqueous foams.

Particles can adsorb at the gas–oil interface and generate “armored” foams in organic solvents, preventing the coalescence of gas bubbles and the drainage of the liquid phase.^13^ For particles to adsorb at the gas–oil interface, they need: (1) overall oleophilicity to disperse in the solvent before foaming; (2) a balanced surface density and distribution of oleophilic and oleophobic (aerophilic) groups to adjust the interfacial contact angle within a stability window;^14^ and (3) controlled size to rapidly diffuse from the bulk liquid to the interface. Few low-surface energy particles can concomitantly meet these three requirements, containing mainly a high surface density of fluorocarbon chains (e.g., fluoropolymers/oligomers,^15−17^ fluorocarbon chains^18−21^) or highly hydrophobic low-carbon chains^22^ distributed in a random fashion. This limited scope arises from the low surface tension of organic solvents restricting particle adsorption at the gas–oil interface.^23^

Janus particles (JPs) are a class of anisotropic colloidal particles that possess different chemical compositions (and opposing wettability) on each hemisphere.^24−28^ This amphiphilic nature allows JPs to adsorb much more strongly at the water–oil interface (up to a 3-fold increase in detachment energy) compared to randomly functionalized counterparts due to much lower liquid–particle surface tensions.^29−32^ As a result, JPs can generate emulsions where interfacial particle self-assembly, orientation, and droplet morphology are governed by the type and density of functional groups on each hemisphere,^33^ and by the particle shape and architecture (e.g., spherical, cubic, icosahedral, nanosheets, dumbbell, mushroom-like).^34−40^ These properties make JPs excellent candidates for engineering biphasic catalytic reactions at the water–oil interface, by carefully locating catalytic centers on either the water or oil sides of the interface.^41,42^ JPs with catalytic centers located on their hydrophobic hemispheres are suitable for reactions in oil phases, including organic synthesis^43−48^ and desulfurization.^49^ In contrast, JPs with catalytic centers located on their hydrophilic hemispheres can promote dye decomposition^50−53^ and photocatalytic water splitting.^54,55^ JPs can also be employed to design interfacial catalysts with spatially isolated acid and basic centers, promoting acid–base tandem reactions.^56^

Despite the significant progress in using JPs to stabilize emulsions, their potential for generating foams has been seldom explored. JPs consisting of a hydrophilic hemisphere incorporating OH/NH_2_ surface groups and an oleophobic hemisphere with either pendant alkyl/fluoroalkyl chains or Au nanoparticles can adsorb at the air–water interface and generate aqueous foams at low particle loadings (<1 wt %).^57−60^ Polymer-functionalized JPs bearing Au nanoparticles can also generate aqueous foams and display a 2.2-fold increase in catalytic activity compared to a nonfoam system in the liquid-phase oxidation of d-glucose to gluconic acid.^61^

Herein, we disclose the high potential of amphiphilic silica JPs with oleophilic and oleophobic hemispheres, featuring selectively spatially located Pd nanoparticles. These particles are capable of generating oil foams using aromatic alcohols at low particle concentrations (1 wt %) with low fluorine content (8 wt % F), and conduct selective aerobic alcohol oxidation reactions at the gas–liquid interface under ambient O_2_ pressure. In this configuration, gas and liquid are expected to directly mix at the gas–liquid interface on the surface of particles by coadsorption, increasing their miscibility and reducing mass transfer resistances. We also report for the first time the direct visualization of the surface distribution of organic moieties on Pd/JPs using photoinduced force microscopy (PiFM), unveiling their anisotropic architecture at the nanoscale level.

## 2 Results and Discussion

### 2.1 Preparation and Characterization of Surface-Active Particles

Pristine silica particles (250 nm) were synthesized using the Stöber method with tetraethyl orthosilicate (TEOS) as the silica precursor, ammonium as the catalyst, and ethanol as the solvent (Figure S1).^62^ JPs (7.89 wt % F) were synthesized by the Pickering emulsion template method,^63,64^ where 1H,1H,2H,2H-perfluorooctyltriethoxysilane (PFOTES) and 3-mercaptopropyltriethoxysilane (MPTES) (equimolar ratio) were sequentially grafted onto silica (Figures S2 and S3, see Supporting Information for details). Randomly functionalized particles (i.e., non-JPs, 8.01 wt % F) were synthesized by concomitant grating of PFOTES and MPTES onto silica. Subsequently, Pd nanoparticles were deposited on both types of particles using a modified sol-immobilization method,^65^ yielding Pd/JPs (0.81 wt % Pd) and Pd/non-JPs (0.82 wt % Pd) (see also Figure S4).

Thermogravimetric analysis (TGA) was used to inspect the stability and grafting efficiency of fluorinated and mercaptoprpyl chains on JPs and non-JPs (Figure S5a). Both types of particles exhibited similar weight loss up to 150 °C (∼1.5%), attributed to water desorption. This weight loss was lower than that measured for pristine silica (∼2.4%) due to the higher hydrophilicity of the latter. The total weight loss of JP and non-JP particles was very similar (19% vs. 18%), indicating the same grafting degree of fluorinated and mercaptopropyl chains (∼8 groups/nm^2^ overall). The derivative TG curves for JPs and non-JPs displayed three main peaks (Figure S5b): (i) a peak at 100 °C that was attributed to water desorption, (ii) a prominent peak at 300–500 °C due to the decomposition of fluorinated and mercaptopropyl chains and ethoxy groups,^66^ and (iii) a peak between 500 and 600 °C that was ascribed to water release due to condensation of SiOH groups.^67^ The derivative curve for the pristine silica also displayed two peaks at 400–500 and 500–600 °C, attributed to the decomposition of ethoxy groups and condensation of silanol groups, respectively.

JPs and non-JPs were analyzed by ^29^Si NMR MAS and ^13^C NMR prior to Pd deposition. The ^29^Si NMR MAS spectrum showed an intense Q_4_ resonance band centered at 111.6 ppm, indicative of siloxane bridges [(SiO)_4_Si] (Figure S6). An intense Q_3_ band was also visible at −102.7 ppm together with a small Q_2_ band at −92.2 ppm, which is ascribed to Si–OH and geminal HO-Si–OH groups. A small T_3_ band was observed at −66.5 ppm, attributed to (SiO−)_3_SiR (tripodal) moieties on silica. Notably, no T^1^ [(SiO−)SiR(−OH)_2_)] (monopodal) and T^2^ [(SiO−)_2_SiR(−OH)] (dipodal) moieties were observed.^68^ The ^13^C NMR MAS spectra of JPs confirmed the grafting of both fluorinated and mercaptopropyl chains (Figure S7). Two bands were observed at 3.8 and 26.6 ppm, attributed to CH_2_ groups in the fluorinated and mercaptopropyl chains, respectively.^69,70^ The bands at 19.6 and 63.5 ppm were indicative of CH_3_ and CH_2_ groups, respectively, in ethoxy groups.^71^ The bands between 110 and 125 ppm were attributed to CF_2_ and CF_3_ groups in the fluorinated chains.^69,70^

**Figure 1:**
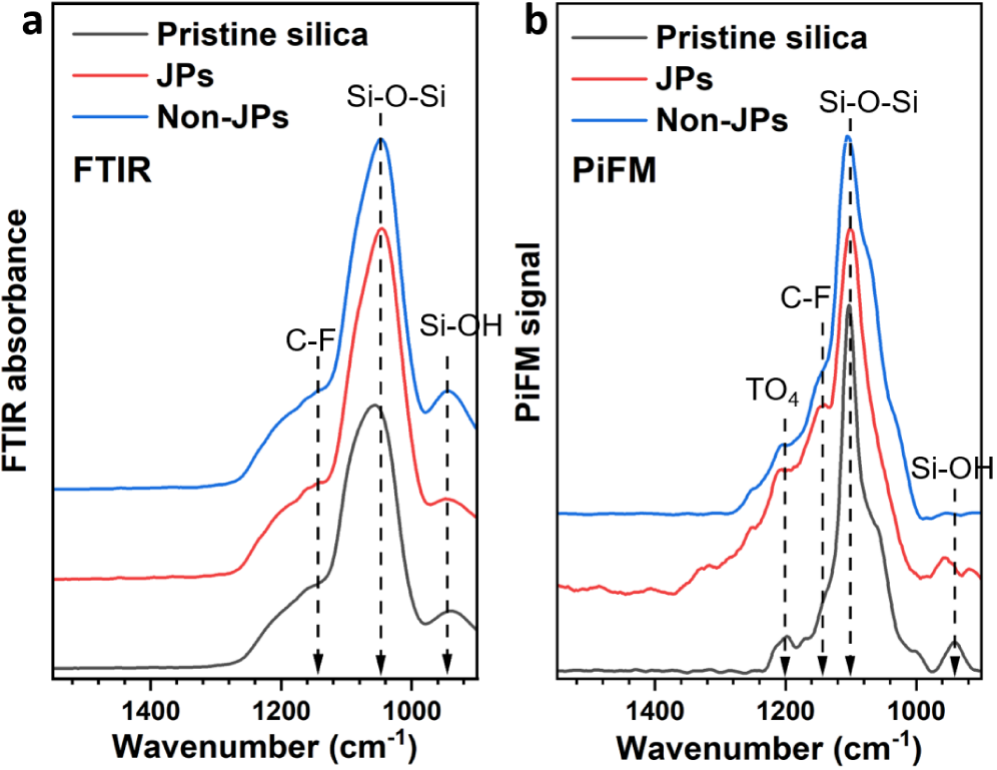
(a) FT-IR and (b) PiFM spectra of pristine silica, JPs, and non-JPs. The green line indicates the location of the C–F stretching vibrations.

The pristine silica, JPs, and non-JPs were further analyzed by FT-IR spectroscopy (Figure 1a and Figure S8). In all cases, two characteristic bands were visible at 1080 and 798 cm^–1^, ascribed to asymmetric stretching and bending vibrations of Si–O–Si bonds, respectively.^68,72^ A large band was also visible in the range 3000–3500 cm^–1^ due to Si–OH groups interacting with adsorbed water. The presence of Si–OH groups was also confirmed by the asymmetric stretching vibration band centered at 950 cm^–1^. A tiny band was visible for non-JPs at 1610 cm^–1^ that can be assigned to asymmetric stretching modes of C–C groups.^73^ No band was observed corresponding to the stretching vibration of S–H groups (2560 cm^–1^),^74^ which can be explained by a low concentration of mercaptopropyl groups. Additionally, there is a broader spectral feature in the 1100–1300 cm^–1^ range associated with weaker transverse (TO) and longitudinal optical (LO) modes. These particular modes, as previously described by Lange et al., are centered at 1254, 1200, and 1170 cm^–1^, representing LO_3_, TO_4_, and LO_4_ modes, respectively.^75^ These weak bands appear as broad shoulders, making it challenging to differentiate them. This difficulty in distinguishing bands becomes apparent when trying to assign the C–F component from PFOTES on functionalized particles as it frequently overlaps with these silica FTIR bands. However, a subtle increase of intensity in the 1150 cm^–1^ region for both JP and non-JP samples hints the presence of C–F bond vibrations.^73^

To gain more insight and resolution on the C–F component, we used PiFM measurements,^76^ taken with a penetration depth of 20 nm in the sideband acquisition mode. The local IR spectra measured by PiFM on different particle locations were congruent with the FT-IR spectra measured on the bulk samples (Figure 1a,b). However, unlike FT-IR, which samples several micrometers into the bulk of the sample and averages over several microns laterally, PiFM mitigates against the interference from bulk SiO_2_ frequencies and thus prevents them from obscuring the C–F component.^73^ For pristine silica surfaces, three prominent IR components were visible at 958, 1095, and 1200 cm^–1^ corresponding to Si–OH, Si–O–Si, and TO_4_ vibrations, respectively (Figure 1b). Upon functionalization with PFOTES and MPTES, the overall intensity of the 1100–1300 cm^–1^ region increased, with notable features at 1225 and 1320 cm^–1^ corresponding to Si-CH_2_ functionalities. The heightened intensity around ∼1150 cm^–1^ confirmed the presence of C–F stretching vibrations on both JPs and non-JPs.

**Figure 2:**
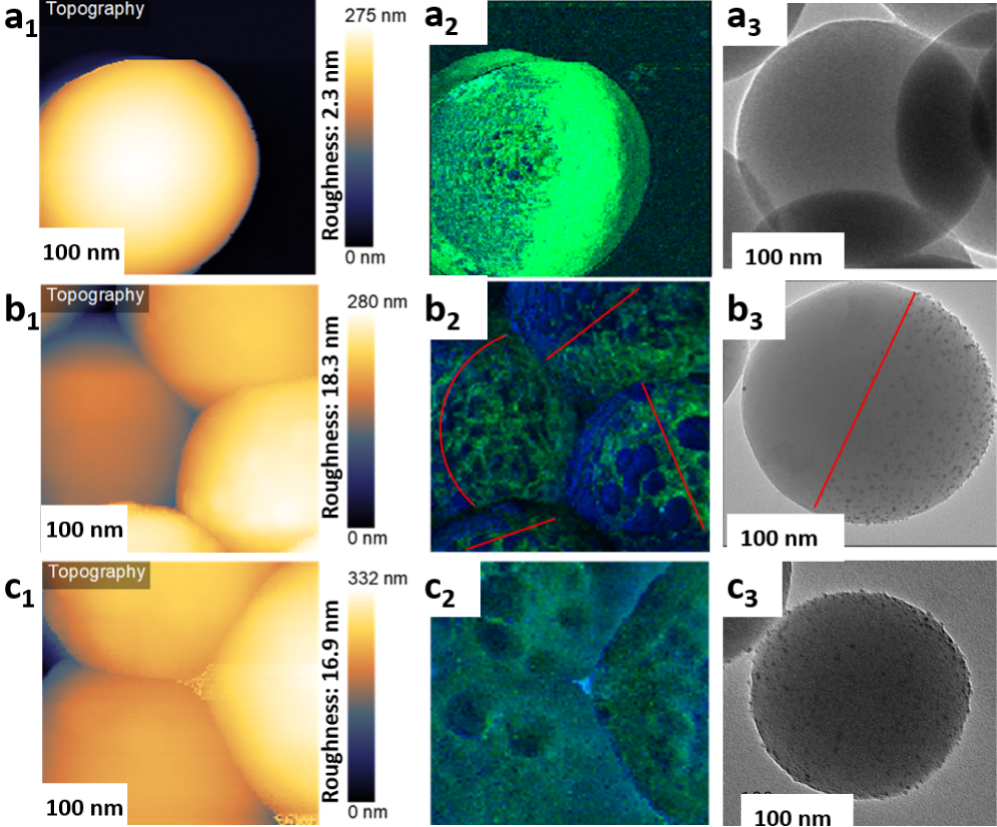
Topography, PiFM at 1145 cm^–1^ and HR-TEM micrographs of (a1–a3) pristine silica, (b1–b3) Pd/JPs, and (c1–c3) Pd/non-JPs. The dark blue and green colors in a_2_–c_2_ refer to fluorocarbon chains and Si–O–Si moieties with bands at 1145 and 1085 cm^–1^, respectively.

The nanoscale distribution of organic moieties on the surface of pristine silica, JPs, and non-JPs was also inspected by PiFM. Pristine particles display relatively smooth surfaces with a roughness of 2.3 nm (Figure 2a_1_). The surface roughness increases to 18.3 and 16.9 nm for JPs and non-JPs, respectively, due to the immobilization of Pd nanoparticles [Figure 2b_1_,c_1_]. The anisotropic nature of JPs is clearly visible in Figure 2b_2_ with fluorohydrocarbon chains (band at 1145 cm^–1^) appearing in dark blue color and the Si–O–Si moieties (band at 1085 cm^–1^) appearing in green (see also Figure 2a_2_). Interestingly, the intensity of fluorohydrocarbon chains is magnified by Pd nanoparticles compared to non-JPs due to a lack of preferential Pd nanoparticle binding in the latter case (Figure 2c_2_). The decrease in the Si component atop Pd nanoparticles points out a spacing between the tip and silica that noticeably affects the acquisition area, given the similar penetration depth of the tip compared to the particle size. Pristine silica, JPs, and non-JPs were also visualized by HR-TEM after loading with Pd (Figure 2a_3_–c_3_). Pd nanoparticles were clearly visible on JPs and non-JPs. Notably, in the case of JPs, Pd nanoparticles were selectively dispersed on the thiol hemisphere (oleophilic).

X-ray photoelectron spectroscopy (XPS) was performed to analyze the Pd speciation on Pd/JPs and Pd/non-JPs (Figure S9a-f). The Pd 3d_5/2_ core level can be deconvoluted into two bands centered at 335.8 and 337.9 eV that can be assigned to Pd(0) and Pd^II^O, respectively (Figure S9b).^77,78^ The C 1s XPS core level region showed bands at 293.3 and 290.9 eV that were assigned to CF_3_ and CF_2_ groups (Figure S9c,d).^79^ The ratio of band areas for the CF_2_ and CF_3_ groups was 4.87, which was consistent with the theoretical value of 5. An additional band was visible at 288.2 eV that was attributed to methylene groups connected with CF_2_.^80^ F 1s and S 2p core level bands were observed at 797.8 and 164.0 eV, respectively, which confirmed the presence of fluorinated and mercaptopropyl chains in Pd/JPs and Pd/non-JPs (Figure S9e,f).

### 2.2 Physicochemical Properties of JPs

**Figure 3:**
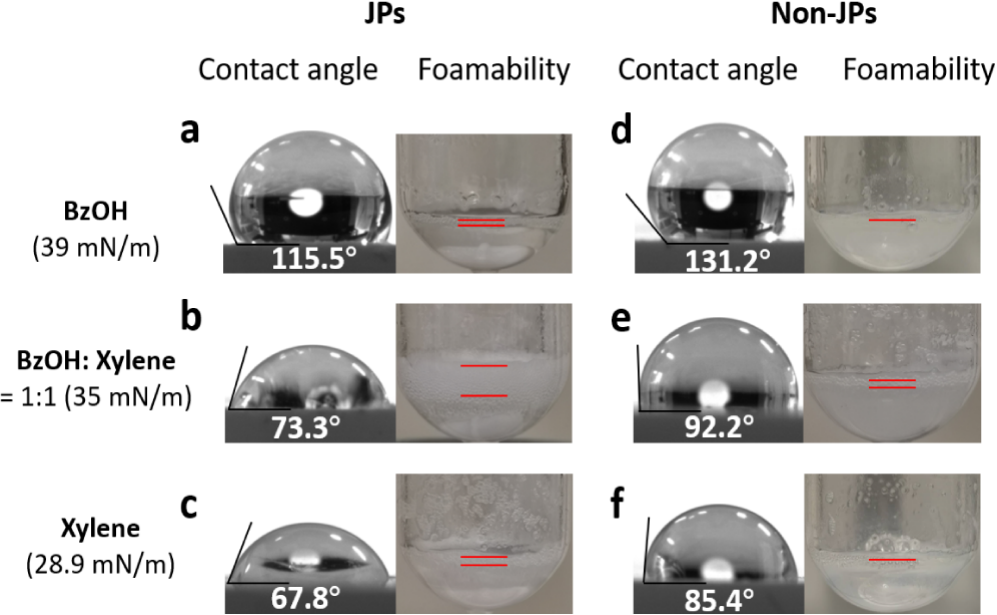
Contact angle and foamability of JPs and non-JPs with (a, d) BnOH, (b, e) mixture, and (c, f) o-xylene. Foaming conditions: 1.8 mL of solvent, 1 wt % particles, 1500 rpm, 100 °C, 1 h.

The interaction of particles with the gas and liquid phases can be characterized by the interfacial contact angle. The contact angles of nonmetal JP and non-JP particles were measured in pure BnOH, pure o-xylene, and in a BnOH–o-xylene (1:1 v/v) mixture. For both particles, the contact angle decreased in the order of BnOH > mixture > o-xylene (see Figure 3a-c for JPs and Figure 3d–f for non-JPs). The contact angles of JPs were systematically lower than those of non-JPs (e.g., 115.5° vs. 131.2°), despite the similar surface density of fluorinated chains, which is consistent with the anisotropic structure of JPs.

**Figure 4:**
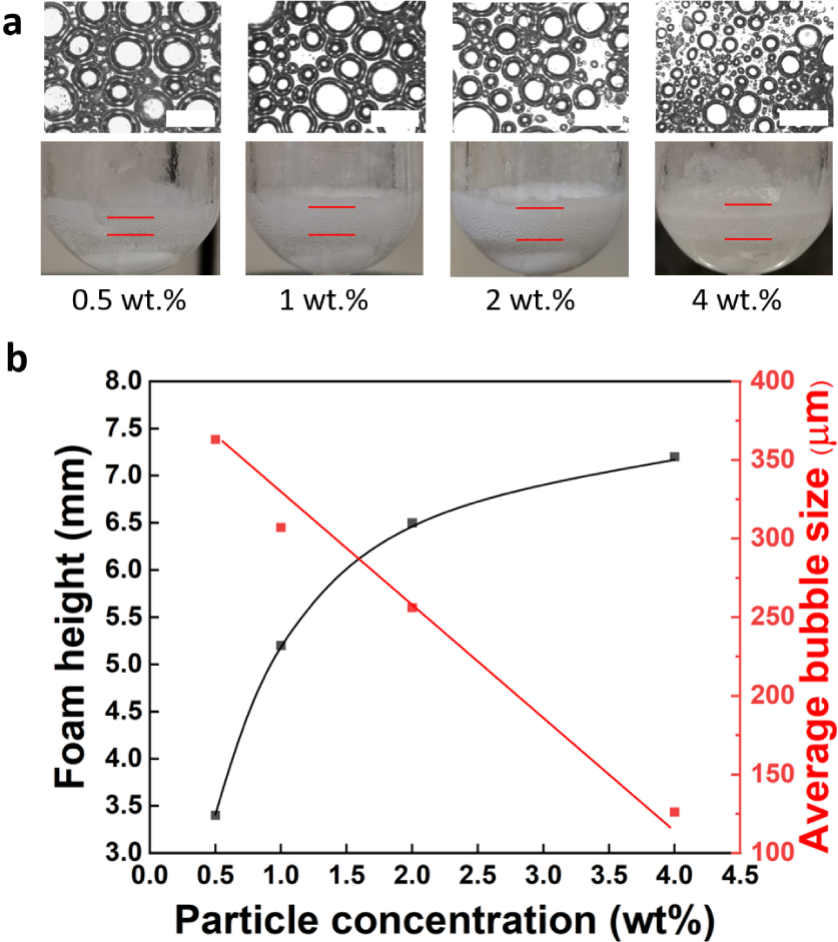
(a) Morphology and foamability of JPs in a BnOH–o-xylene (1:1 v/v) mixture at room temperature and variable JP loading. The bar size is 500 μm. (b) Evolution of the foam height (left ordinate axis) and average bubble (right ordinate axis) against the particle concentration. Foaming conditions: 0.8 mL of BnOH, 0.8 mL of o-xylene, 0.5–4.0 wt % JPs, 1500 rpm, 100 °C, 1 h.

**Figure 5:**
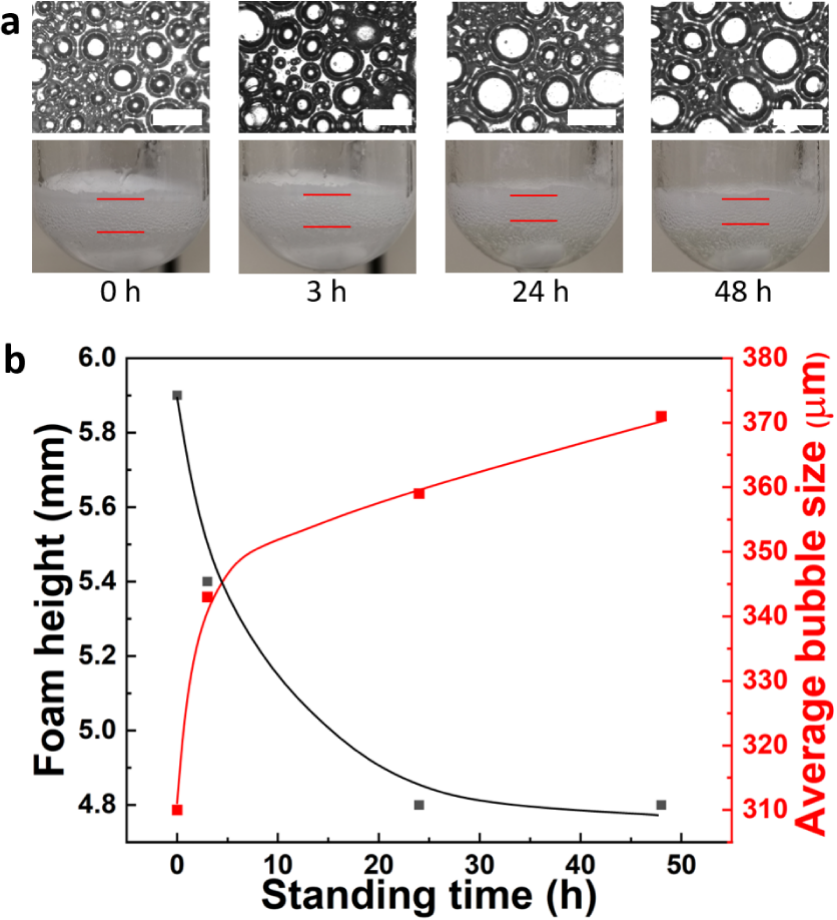
(a) Morphology and foam stability of JPs in BnOH–o-xylene (1:1 v/v) mixture at room temperature and 1 wt % JP loading. The space bar size is 500 μm. (b) Time evolution of foam height and average bubble size. Foaming conditions: 0.8 mL of BnOH, 0.8 mL of o-xylene, 1 wt % JPs, 1500 rpm, 100 °C, 1 h, kept static at room temperature.

The foamability of metal-free JPs and non-JPs was studied in pure BnOH, pure o-xylene, and in a BnOH–o-xylene (1:1 v/v) mixture at 100 °C with a particle loading of 1 wt % and a stirring rate of 1500 rpm. Both particles display no foamability in pure BnOH (surface tension = 39 mN m^–1^) and o-xylene (surface tension = 28.9 mN m^–1^). In contrast, JPs exhibit excellent foamability in a BnOH–o-xylene (1:1 v/v) mixture (surface tension = 35 mN m^–1^), whereas only a very thin layer is formed using non-JPs. These results point out a much stronger interfacial adsorption of JPs compared to non-JPs in a BnOH–o-xylene (1:1 v/v) mixture. Notably, JPs exhibit foaming properties comparable to those of fluorinated silica particles prepared by coprecipitation, which require much higher fluorine content (25–33 wt % vs. 8 wt % for JPs).^81^ Loading of JPs and non-JPs with Pd nanoparticles does not alter their foamability (Figure S10a). The foamability (i.e., foam height) of JPs increases dramatically from 3.2 to 7.2 mm when the particle loading is increased from 0.5 to 4 wt %, with a concomitant decrease in the average bubble size from 355 to 115 μm (Figure 4). The foams show high stability for at least 48 h, with a minor decrease in foam height from 5.9 to 4.8 mm and an increase in the average droplet size from 305 to 370 μm (1 wt % JP loading) (Figure 5). The bubble size distributions are collected in Figure S11. The liquid phase turns turbid immediately after foaming, revealing that a small fraction of particles do not adsorb at the gas–liquid interface. These particles sediment further within 3 h, leading to a clear liquid phase.

The dispersion of JPs and non-JPs in a BnOH–o-xylene mixture (1:1 v/v) was investigated by dynamic light scattering (DLS). Non-JPs display agglomeration even at very low concentrations (0.001 wt %) with an average particle size of 1280 nm, which becomes more prominent at higher particle concentrations (0.01 and 0.1 wt %). The agglomeration of non-JPs is systematically larger compared to JPs, which is consistent with the higher hydrophobicity of the former, as inferred from their higher contact angles (Figure S12). The broader peak for non-JPs reveals a higher polydispersity that can be explained by their higher hydrophobicity compared to JPs, making them more difficult to wet with the BnOH–xylene mixture.

To assess the surface activity of JPs and non-JPs at the gas–liquid interface, the surface tension of the BnOH–o-xylene mixture (1:1 v/v) was measured before and after adding JPs and non-JPs (0.001, 0.01, and 0.1 wt %) (Figure S13). The results demonstrated that both particles cannot reduce the surface tension, which is consistent with previous reports.^82^

### 2.3 Aerobic Oxidation of BnOH

**Figure 6:**
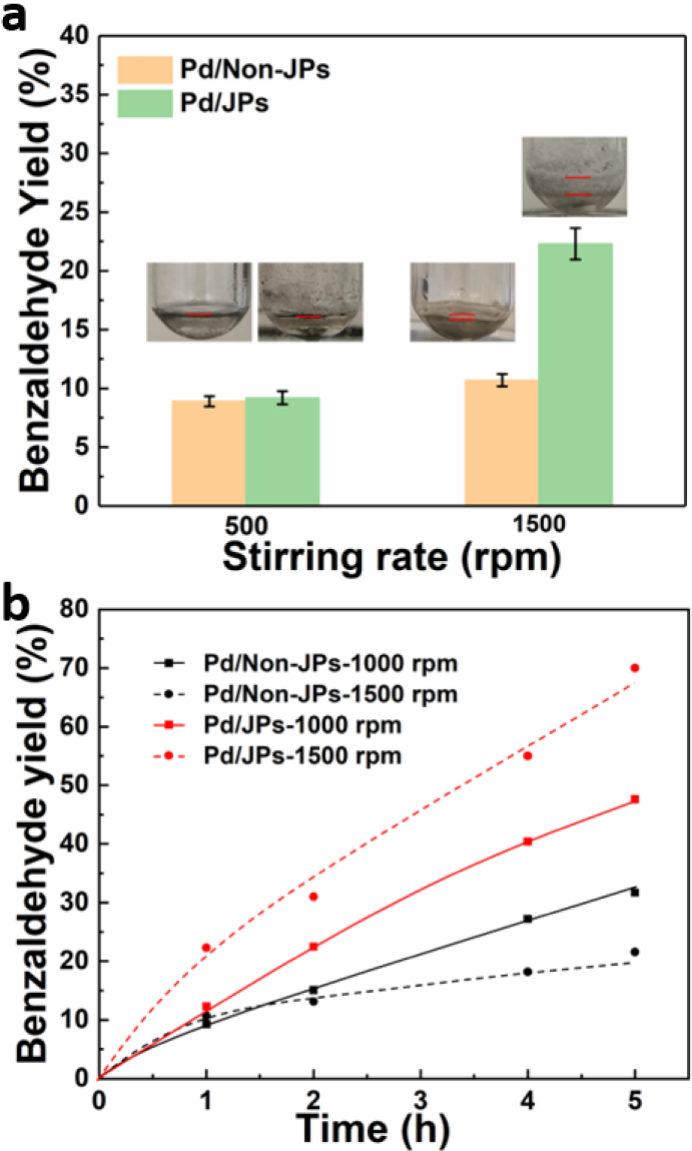
(a) Aerobic oxidation of BnOH over Pd/JPs and Pd/non-JPs. Reaction conditions: 0.8 mL of BnOH, 0.8 mL of o-xylene, O_2_ balloon, 1 wt % particles, 1500 rpm, 100 °C, 1 h. (b) Kinetic profiles for the aerobic oxidation of BnOH over Pd/JPs and Pd/non-JPs. Reaction conditions: 0.8 mL of BnOH, 0.8 mL of o-xylene, an O_2_ balloon, 1 wt % particles, 1000 and 1500 rpm, 100 °C, variable reaction time.

Based on the aforementioned results, we investigated the catalytic properties of Pd/JPs and Pd/non-JPs (1 wt %) in the aerobic oxidation of BnOH in a BnOH–o-xylene (1:1 v/v) mixture with and without foam (Figure 6a). The reaction was conducted at 100 °C for 1 h using stirring rates of 500 and 1500 rpm. Under nonfoaming conditions (500 rpm), both particles exhibited a similar benzaldehyde (BAH) yield (about 9%). However, at 1500 rpm, Pd/JPs displayed a prominent increase in the BAH yield (22%), whereas it remained almost unchanged (∼9%) for Pd/non-JPs. The marked difference in catalytic activity between the two particles is attributed to the abundant foam generation by Pd/JPs at 1500 rpm, whereas Pd/non-JPs display low foamability at the same stirring rate. Figure S14 illustrates the evolution of BnOH conversion and selectivity to different oxidation products in the aerobic oxidation reaction of BnOH over Pd/JPs and Pd/non-JPs with and without foam as a function of stirring speed. For Pd/JP particles, BnOH conversion increases from 10% to 25% as the stirring speed is raised from 500 to 1500 rpm. Meanwhile, the BAH selectivity increases from 86% to 92%, which can be explained by higher O_2_ accessibility to the active sites, concomitantly decreasing toluene selectivity due to BnOH disproportionation from 11% to 7%. Opposing these observations, the BnOH conversion increases only slightly (from 10% to 12%) for Pd/non-JPs when the stirring speed is raised from 500 to 1500 rpm, whereas the BAH and toluene selectivities remain almost unchanged at 11%. For this catalyst, BnOH conversion increases from 12% to 17% when raising the O_2_ pressure from 1 to 5 bar (maximum pressure of our reactor) (Figure S15). To reach the BnOH conversion obtained using Pd/JPs in foam at 1500 rpm (25%), the reaction requires a much higher O_2_ pressure, whereas the reaction in foam is operated at ambient pressure. In all cases, no benzoic acid was observed (selectivity <1%), which can be explained by radical scavenging from BnOH.^83^ Benzyl benzoate was formed in very small amounts (selectivity <1%), which may come from a hemiacetal intermediate that is expected to be unstable under the reaction conditions and oxidized to the ester.^84^

The kinetic profiles were measured for Pd/JPs and Pd/non-JPs at 100 °C in a BnOH–o-xylene (1:1 v/v) mixture at at stirring speeds of 1000 and 1500 rpm (Figure 6b). The BAH yield increases faster and reaches higher values after 5 h reaction over Pd/JPs compared to Pd/non-JPs due to the formation of foam. Increasing the stirring speed from 1000 to 1500 rpm results in a prominent increase in activity and final yield due to enhanced foamability. It should be noted that the BAH yield obtained over Pd/non-JPs at 1500 rpm is lower than that measured at 1000 rpm after 2 h, which can be attributed to partial catalyst adherence to the reactor wall that decreased the amount of the available catalyst for the reaction. This phenomenon was not observed at 1000 rpm. From the kinetic plots, the catalytic activity at time = 0 (turnover frequency, TOF_0_) was 2118 h^–1^ for Pd/JPs and 1046 h^–1^ for Pd/non-JPs at 1500 rpm. The TOF_0_ remained unchanged for non-JPs when changing the stirring speed from 1000 to 1500 rpm. As a result, the BAH yield reached 70% for Pd/JP after 5 h at 1500 rpm, whereas it was only 32% for Pd/non-JPs at 1000 rpm (used as a reference). We measured the activation energies (*E*_a_) for Pd/JPs and Pd/non-JPs from the Arrhenius plots of TOF values in the temperature range of 353–383 K (Figure S16). The activation energy was 103 kJ/mol for Pd/non-JPs in the bulk system and decreased to 86 kJ/mol for Pd/JPs in the foam system. The higher activation energy in the former case was consistent with the observation reported earlier for alcohol oxidation in aqueous foams,^85^ and also aligned with reported results pointing out that the activation energy of oxidation occurring at a gas–solid interface was meaningfully different from that at the liquid–solid interface.^86^ As a matter of fact, in Pd/non-JPs, the oxidation reaction occurs between the alcohol and dissolved O_2_, whereas in Pd/JPs, the reaction mainly occurs at the gas–liquid–solid interface, which alters the local microenvironment and thereby modifies the reaction mechanism on the catalyst surface, decreasing the apparent activation energy and consequently increasing the reaction rate.

Overall, we can infer from these results that the reaction is conducted in the absence of mass transfer resistances for Pd/non-JPs, as the catalytic activity remains unchanged with stirring speed. In the case of the Pd/JPs foaming system, the catalytic activity and selectivity increase with stirring speed, which is attributed to enhanced gas–liquid–catalyst contact at the surface of bubbles.

### 2.4 Catalyst Recyclability and Reuse

**Figure 7:**
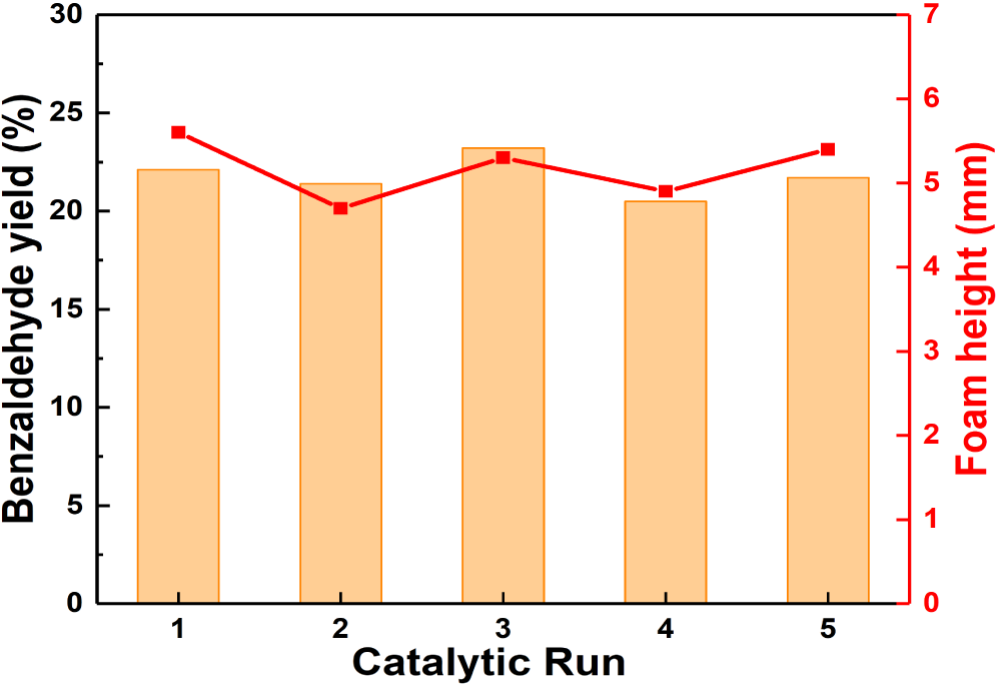
Recyclability and reuse of Pd/JPs for the aerobic oxidation of BnOH over five consecutive runs. Reaction conditions: 0.8 mL of BnOH, 0.8 mL of o-xylene, 1 wt % Pd/JPs, 1 bar O_2_ balloon, 1500 rpm, 100 °C, 1 h.

We further studied the recyclability and reuse of Pd/JPs in the aerobic oxidation of BnOH for five consecutive runs. The catalytic tests were carried out at 100 °C for 1 h using 1 wt % Pd/JPs. After each run, the catalytic particles were separated by centrifugation at 7200 rpm for 3 min, washed twice in acetone, and dried at 80 °C for 4 h before reuse in the subsequent run. Pd/JPs retained their robust activity and foamability after each run without any significant loss (Figure 7). The reaction selectivity remained unchanged after recycling, as shown in Figure S17. No evidence of Pd leaching during the reaction was observed, as inferred from ICP-MS analysis of the catalysts after the fifth run (Table S2). Also, no Pd sintering was observed after the fifth run by comparing the size distribution of Pd nanoparticles on Pd/JPs before and after the reaction (Figure S4a-d). This observation can be attributed to the thiol groups acting as anchors, providing stability to Pd nanoparticles.^87^

### 2.5 Extension to Aromatic and Aliphatic Alcohols

The aforementioned results point out significantly higher catalytic activity of Pd/JPs compared to Pd/non-JPs for the aerobic oxidation of BnOH at comparable grafting degree. We then used Pd/JPs to conceive foam systems for the aerobic oxidation of aromatic alcohols under O_2_ using 1 wt % particles (Table 1). In particular, we designed stable foam systems for cinnamyl alcohol–o-xylene, 1-phenylethanol–dodecane, 2-phenylethanol–dodecane, and vanillyl alcohol–dodecane mixtures, all at 1:1 v/v ratios (Figure S10b-e). Cinnamyl alcohol can be oxidized to cinnamaldehyde at 100 °C for 1 h over Pd/JPs in foam with 34% yield (entry 2), whereas the yield is only 13% over Pd/non-JPs in a nonfoaming system. In the case of 1-phenylethanol, the foam system stabilized by Pd/JPs yields 6.6% phenylacetaldehyde at 120 °C after 1 h of reaction, whereas the nonfoam system (Pd/non-JPs) yields only 2.1% under the same conditions (entry 3). Increasing the temperature to 140 °C raises the yield in the nonfoam system to 5.4%, which is still lower than the yield in the foam system at 120 °C. The 2-fold increase in yield is a result of the elevated kinetic energy associated with the temperature rise from 120 to 140 °C. Likewise, when converting 2-phenylethanol to acetophenone, the foam system stabilized by Pd/JPs affords 14% yield at 120 °C after 1 h, while the yield is only 2.1% over Pd/non-JPs in a nonfoam system (entry 4). Finally, vanillyl alcohol is converted into vanillin with 34% yield at 120 °C after 1 h over Pd/JPs in a foam system, while the yield only reaches 16% over Pd/non-JPs in a nonfoam system. Furthermore, we broadened the scope from aromatic alcohols to aliphatic alcohols. Pd/JPs generated abundant foam in 1-octanol and 2-octanol, resulting in higher yields for aerobic oxidation, reaching 19.7% and 4.4%, respectively, compared to the Pd/non-JP system without foam, where the yields were 4.4% and 1.4%.

**Table 1:**
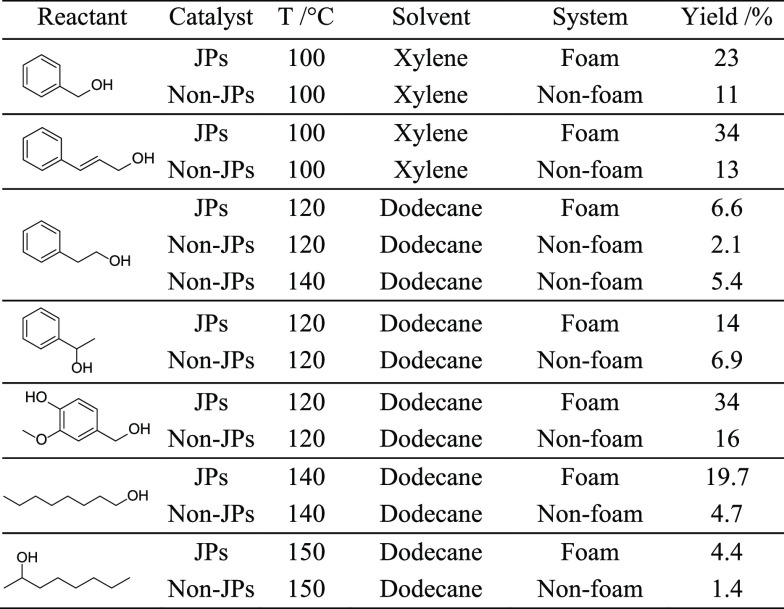
Substrate Scope Expansion for Catalytic Tests in O_2_ Atmosphere^a^

a Reaction conditions: 0.8 mL substrate, 0.8 mL solvent, 1 wt % Pd/JP catalysts, 1500 rpm, O_2_ balloon, 1 h. (According to the Antoine equation, the partial pressure correction of O_2_ at 100 °C in the solvent of o-xylene is approximately 0.74 bar. The partial pressure in the solvent of dodecane at 120, 140, and 150 °C are 0.95, 0.89, and 0.85 bar, respectively).

## 3 Conclusions

In summary, we prepared silica Janus particles grafted selectively with fluorinated and mercaptopropyl chains on each hemisphere, enabling tunable design of oleophobic–oleohipilic properties with low fluorine content (8 wt % F). The particles were decorated with Pd nanoparticles in the oleophilic hemisphere. The anisotropic surface architecture of these particles was confirmed using photoinduced force microscopy, which provided high-resolution imaging of fluorocarbon chains near the Pd nanoparticles. Janus particles exhibited higher foamability in oil solvents compared to particles with a homogeneous surface distribution of fluorinated and mercaptopropyl chains at the same surface density of fluorinated and mercaptopropyl groups owing to their stronger adsorption at the oil–O_2_ interface. The catalytic performance was strongly affected by the foaming properties, with Pd-loaded Janus particles exhibiting at least double yield of aldehyde/ketone products in the aerobic oxidation of aromatic alcohols compared to non-Janus particles. The extension of aromatic and aliphatic alcohols in the foam system confirms the generality of the JP foam system. Janus particles were conveniently recycled with high foamability and catalytic efficiency maintained for at least five consecutive runs.

The results presented in this study pave the way for designing purposeful and adjustable oleophobic–oleophilic Janus particles to generate oil foams *à la carte* for a large variety of aromatic alcohols. This approach could be extended to other alcohols and organic reactants by fine design of particle hemispheres and nanoscale distribution of organic functions.

## Supporting Information Available

Particle size distribution of different particles; size distribution of Pd nanoparticles; TGA profiles, ^13^C NMR MAS and XPS spectra and HR-TEM images of different particles; optical images of different kinds of aromatic alcohols in xylene foams at 100 °C after reaction; PiFM topography and spectroscopy of Janus nanoparticles. DLS results about different particles in BnOH and xylene mixture; surface tension tests of different particles in the mixture of BnOH and o-xylene (PDF)
