# Supplementary material for: Amphiphilic Janus Particles for Aerobic Alcohol Oxidation in Oil Foams
Source: ACS Catal. 2024 Jul 19;14(15):11545–53. doi: 10.1021/acscatal.4c00909 (PMC11301628; doi:10.1021/acscatal.4c00909)
Supplement: Supplementary file 1 — cs4c00909_si_001.pdf [file cs4c00909_si_001.pdf]

Supporting materials

Amphiphilic Janus Particles for Aerobic Alcohol Oxidation in Oil Foams

Kang Wang,[1] Josh Davies-Jones,[1] Arthur Graf,[1] Marina Carravetta,[2] Philip R. Davies,[1] Marc Pera-Titus[1*]

[1] Cardiff Catalysis Institute, School of Chemistry, Cardiff University, Main Building, Park Place, Cardiff CF10 3AT, UK

[2] School of Chemistry, University of Southampton, Highfield, SO17 1BJ, Southampton, UK

Corresponding Author. E-mail: peratitusm@cardiff.ac.uk

Table of Contents

Figure S1. (a) Dynamic light scattering (DLS) result for pristine silica particles. (b) Scanning electron microscope (SEM) image of pristine silica particles. (c) Particle size distribution of Pristine silica.

Figure S2. SEM image of the Stöber silica particles embeds in the wax droplet in half.

Figure S3. General scheme of the synthesis protocol of JPs.

Figure S4. Size distribution of Pd nanoparticles in (a) fresh Pd/Non-JPs, (b) fresh Pd/JPs, (c) spent Pd/JPs after five consecutive catalytic runs, and (d) TEM image of spent Pd/JPs after five consecutive catalytic runs.

Figure S5. (a) TG and (b) DTG profiles of pristine silica, JPs and Non-JPs in air atmosphere.

Figure S6. Solid-state ^{29}Si NMR MAS spectra of (a) JPs and (b) Non-JPs.

Figure S7. Solid-state ^{13}C NMR MAS spectra of JPs and Non-JPs.

Figure S8. FT-IR spectroscopy of pristine silica, JPs and Non-JPs.

Figure S9. (a) X-ray photoelectron spectroscopy (XPS) survey spectrum of Pd/JPs and Pd/Non-JPs surface. (b) High-resolution narrow scan of the two particles for Pd(3d). (c) Molecular structure of the silanes grafted on the surface of silica particles. (d) High-resolution deconvoluted spectra of the two particles for C(1s). (e) High-resolution narrow scan of the two particles for F(1s). (f) High-resolution narrow scan of the two particles for S(2p).

Figure S10. Foamability of Pd/JPs and Pd/Non-JPs in different solvents; (a) Benzyl alcohol, (b) 1-phenyl ethanol, (c) 2-phenyl ethanol, (d) Vanillyl alcohol, (e) Cinnamyl alcohol. Conditions: 0.8 mL of aromatic alcohol, 0.8 mL of o-xylene or dodecane, 1 wt % particle, O_2 balloon, 1500 rpm, 100-120 °C, 1 h.

Figure S11. Bubble size distribution for foams prepared with JPs at variable weight loading. Foaming conditions: 0.8 mL of BnOH, 0.8 mL of o-xylene, 0.5-4.0wt% JPs, 1500 rpm, 100 °C, 1 h.

Figure S12. Size distributions for JPs and Non-JPs at 0.001 wt%, 0.01 wt%, and 0.1 wt% in BnOH/o-xylene mixture (1:1 v/v) measured by DLS.

Figure S13. Surface tension of BnOH/o-xylene mixture (1:1 v/v) with the concentration of 0.001 wt%, 0.01 wt%, and 0.1 wt% JPs and Non-JPs.

Figure S14. BnOH conversion and selectivity to different products in the aerobic oxidation of BnOH over Pd/JPs and Pd/ Non-JPs with and without foam, respectively. Reaction conditions: 0.8 mL of BnOH, 0.8 mL of o-xylene, 1wt% catalysts, 500-1500 rpm, 100 °C, 1 h.

Figure S15. BnOH conversion and selectivity to different products in the aerobic oxidation of BnOH over Pd/Non-JPs with different 1 and 5 bar O_2 pressure. Reaction conditions: 0.8 mL of BnOH, 0.8 mL of o-xylene, 1wt% Pd/Non-JPs, 1500 rpm, 100 °C, 1 h.

Figure S16. Kinetic plots of –Ln(1-C) against time for the aerobic oxidation of BnOH catalyzed by (a) Pd/Non-JPs (without foam) and (b) Pd/JPs (with foam); (c) Variation of TOF at time t=0 for with reciprocal temperature for Pd/JPs and non-JPs.

Figure S17. BnOH conversion and selectivity to the different products in the aerobic oxidation of BnOH over Pd/JPs in foam in five consecutive runs. Reaction conditions: 0.8 mL of BnOH, 0.8 mL of o-xylene, 1wt% Pd/JPs, 1500 rpm, 100 °C, 1 h.

Table S1. Pd concentration in the catalysts before and after reaction (measured by ICP-MS)

Experimental Procedures

Materials

Tetraethyl orthosilicate (TEOS, 98%), N-cetytrimethylammonium bromide (CTAB, 98%), 1H,1H,2H,2H-perfluorooctyltriethoxysilane (PFOTES, 97%), (3-mercaptopropyl)triethoxysilane (MPTES, >80%), ammonium hydroxide solution (28-30%), anhydrous ethanol (99.9%), palladium(II) chloride (98%), sodium borohydride ($NaBH_4$, 98%), Poly(vinyl alcohol) (PVA, Sigma-Aldrich, 80% hydrolyzed), all purchased from Sigma-Aldrich, were used for the synthesis of catalytic silica particles. Benzyl alcohol (>99%), benzaldehyde (>99%), o-xylene (reagent grade), acetone (>99.5%), dodecane (>99%), 4-methylbenzyl alcohol (98%), 2-phenylethanol (98%), phenylacetaldehyde (98%), 1-phenylethanol (98%), acetophenone (99%), vanillyl alcohol (>98%), vanillin (99%), cinnamyl alcohol (98%) and cinnamaldehyde (>95%), also supplied by Sigma-Aldrich, were used for the oxidation reactions.

Methods

Preparation of silica nanoparticles

The synthesis of silica particles was carried out by the Stöber method, whereby a mixture of 15 mL of water, 50 mL of ethanol and 3 mL of 35% of an aqueous ammonia solution was heated to 25 °C. Subsequently, 6 mL of 99% TEOS dissolved in 50 mL ethanol was added to this solution and rapidly stirred at a rate of 1500 rpm for 1 min to form the silica seeds. The reaction was then allowed to proceed for 60 min at 500 rpm. The resulting silica spherical particles were separated by centrifugation and washed three times with ethanol and water before drying at 110 °C in air overnight. The particles are denoted as parent silica.

Preparation of catalytic silica nanoparticles

Preparation of Janus particles. Initially, 400 mg of the parent silica particles were dispersed in 30 mL of 0.45 mM CTAB solution, followed by the addition of 4.5 g of paraffin wax at 75°C at constant stirring until all the wax was completely melted. The resulting emulsion was formed using Ultra-turrax at 30,000 rpm for 1 minute, followed by cooling to room temperature, causing the paraffin wax to solidify. Subsequently, the wax emulsions were washed with deionized water multiple times to eliminate any particles in the aqueous phase, weakly attached particles, and CTAB molecules. The silica embedded within the wax droplets was then observed using SEM, as depicted in **Figure S2**. The particles adsorbed on the wax were reacted with 2 mM (3-mercaptopropyl)triethoxysilane in 30 mL deionized water for 3 h at room temperature to modify the exposed surfaces. The resulting wax droplets were filtered with medium-speed filter paper, washed on the filter with 500 mL deionized water, and dried at 25 °C overnight. Subsequently, the wax was dissolved in cyclohexane, and the particles were washed 3 times with 40 mL cyclohexane by centrifugation at the speed of 4300 rpm and dried at 110 °C overnight. Further modification of the resulting particles was carried out by treating the left hemisphere with 2 mM 1H,1H,2H,2H-perfluorooctyltriethoxysilane in 10 mL toluene at 110 °C. The particles were then washed 3 times with 40 mL ethanol by centrifugation at 4300 rpm and dried at 110 °C overnight. The particles were denoted as JPs.

Loading of Pd nanoparticles. $PdCl_2$ (10 mg/mL) and PVA (Pd/PVA = 1: 1 wt/wt) solutions were added to 15 mL of water. After stirring for 10 min, a 0.1 mM aqueous solution of $NaBH_4$ ($Pd/NaBH_4$ = 1: 5 mol mol^{-1}) was added to the yellow solution under vigorous magnetic stirring. A black Pd(0) sol was immediately formed. Within a few minutes from their generation, the colloids (acidified at pH 2 by sulfuric acid) were immobilized by adding the above particles dispersed in 5 mL ethanol under stirring. After 2 h of aging, the dispersion was filtered and washed with 1 L of deionized water. The catalyst was dried at 110 °C overnight. The protocol is schematized in **Figure S3**.

Preparation of Non-JPs. 300 mg of pristine silica particles were dispersed in 10 mL toluene. 2 mM (3-mercaptopropyl)triethoxysilane and 1H,1H,2H,2H-perfluorooctyltriethoxysilane were added into the above dispersion. The mixture was heated at 110 °C for 2 hours. The particles were then washed 3 times with ethanol by centrifugation and dried at 110 °C overnight. The particles were denoted as Non-JPs.

Loading of Pd nanoparticles. $PdCl_2$ (10 mg/mL) and PVA (Pd/PVA = 1: 1 wt/wt) solutions were added to 15 mL of water. After stirring for 10 min, a 0.1 mM aqueous solution of $NaBH_4$ (Pd/$NaBH_4$ = 1: 5 mol mol^{-1}) was added to the yellow solution under vigorous magnetic stirring. A black Pd(0) sol was immediately formed. Within a few minutes from their generation, the colloids (acidified at pH 2 by sulfuric acid) were immobilized by adding the above particles dispersed in 5 mL ethanol under stirring. After 2 h of aging, the dispersion was filtered and washed with 1 L of deionized water. The catalyst was dried at 110 °C overnight. The protocol is schematized in **Figure S3**.

Catalyst characterization

The thermal profiles of the different particles were measured on a TGA instrument. The particles (∼10 mg in a 100 µl alumina crucible) were treated from 30 to 900 °C with a heating rate of 10 °C/min under airflow of 30 mL(STP)/min.

The Pd composition of the catalysts was analyzed by Inductively Coupled Plasma Mass Spectrometry (ICP-MS) on an Agilent 7900 ICP-MS instrument equipped with an I-AS auto-sampler using five-point calibration, certified reference materials from Perkin Elmer and a certified internal standard from Agilent. All reference materials were matrix-matched to increase the accuracy of quantitative analyses. The samples (20 mg) were weighed and put in the ICP tube with 8 mL of aqueous HNO_3 solution. The tube was treated in a microwave oven at 220 °C for 30 min. After cooling down to room temperature, the samples were treated with 50 mL of deionized water for dilution.

The surface composition of the catalysts was analyzed by X-ray photoelectron spectroscopy (XPS) on a Kratos Axis Ultra DLD spectrometer with monochromatic Al Kα radiation (hν = 1486.6 eV). Samples were mounted using double-sided adhesive tape, and binding energies were referenced to the C(1s) binding energy of adventitious carbon contamination taken to be 284.8 eV. The spectra were recorded using a pass energy of 160 eV for survey scans, while 40 eV was employed for detailed regional scans. CasaXPS v2.3.24 using a Shirley background and modified Wagner elemental sensitivity factors as supplied by the instrument manufacturer.

Scanning electron microscopy (SEM) imaging of particles adsorbed in wax was performed on Tescan Maia3 FEG-SEM microscope. The samples were coated with 15-nm Au thickness on the surface to increase the conductivity. The operation voltage was 15 kV in the mode of in-beam BSE.

The particle size distributions of silica particles and Pd nanoparticles were measured by transmission electron microscopy (TEM) on a JEOL JEM-2100 operating at 200 kV. Samples were prepared by dry deposition onto 300 mesh copper grids coated with holey carbon film. The images were analyzed by ImageJ software. At least 100 particles were counted for the statistic chart. In the analyses, we assumed that the metal particles are spherical in shape and we took explicitly into account the density ratio between the oxide and metal phases. The Pd dispersion of Pd nanoparticles, D_{Pd}, was measured from the corresponding average particle size according to the expressions provided by Borodzinski and Bonarowska.[S1]

The Fourier-transformed infrared spectra (FT-IR) of the silica particles were measured from 500-4000 cm^{-1} on a Bruker Tensor 27 spectrometer equipped with a HgCdTe (MCT) detector and operated with OPUS software. Each spectrum was measured after 256 scans.

Solid-state ^{13}C and ^{29}Si NMR MAS spectra were acquired on a Bruker AVANCE III 500 spectrometer with a wide bore 11.7 Tesla magnet with operational frequencies for ^{13}C and ^{29}Si of 500 MHz. A 4-mm triple resonance probe in double resonance mode with magic angle spinning (MAS) was employed in all the experiments, and the samples were packed on a zirconia rotor and spun at a MAS rate of 15 kHz. The radio frequency field was 11 kHz for both ^{13}C and ^{29}Si. The direct acquisition is the result of 256 scans with a pulse delay of 6 min (one day acquisition). Cross Polarization (CP) was performed with 2000 scans. In the case of ^{13}C and ^{29}Si MAS NMR, cross polarization for proton decoupling was applied. The relaxation delay, d1, between accumulations was 5, 1 and 60 s for ^{13}C and ^{29}Si MAS NMR spectroscopy, respectively. All chemical shifts were reported using δ scale, and were externally referenced to glycine for ^{13}C NMR, TMS for ^{29}Si NMR. The samples were packed into an NMR rotor and dehydrated at 573 K under vacuum ($1x10^{-4}$ mbar) for 2 h

prior to the loading into the magnet and recording of the NMR spectrum.

Photo-induced force microscopy (PiFM) Imaging and spectral data were acquired using a Vista One nano-IR microscope & spectrometer (Molecular Vista Inc, USA) equipped with a Bloc 780-1930 cm^{-1} quantum-cascade laser (QCL) and Vistascan Version 28 (Molecular Vista Inc, USA). Non-contact high-resolution PtIr-coated (NCHR) cantilevers were used in the measurements. These cantilevers were initially sourced from Molecular Vista (Molecular Vista Inc, USA) and had a spring constant of 45 n/M and a resonance frequency of 335 kHz. All measurements were conducted in Sideband mode, providing a 20 nm penetration depth, with a spectral resolution of 1 cm^{-1}. Subsequently, both spectra and images were subjected to analysis using Surfaceworks 3.0 Release 32 (Molecular Vista Inc, USA). The particle samples were prepared by sprinkling 100 mg of particles onto a steel disc coated with epoxy resin and allowed to be set over 48 hours. The samples were blown with dry air to remove all excess particles prior to analysis.

Interfacial contact angles were measured by the sessile drop method (performed with a Dataphysics OCA 35 device) by depositing a small drop (repeated three times per sample) of 8 µL of liquid on pellets formed from powder. The pellets were prepared using at least 200 mg particle powder and then subjected to compression under a load of 3 tons using a press for 5 min. The shape of the drops was observed and used to determine the contact angles.

Surface tension measurements of benzyl alcohol (BnOH) / o-xylene mixtures with/without particles were carried out using a TD4 Lauda scientific tensiometer mounted on a Du Noüy ring with 9.75 mm radius and 0.2 mm wire radius. 5 mL of o-xylene and 5 mL BnOH were added into a 50 mL glass vessel. At the start of the measurements, the ring was pulled out of the mixed solvents while measuring the force. When the maximum force was reached, the movement direction of the platform changed and brought the ring back to the mixture. This process was repeated until the set standard deviation was reached. The results were recorded and repeated 3 times for each test.

Catalytic tests.

The oxidation of BnOH in o-xylene was conducted in a batch reactor under pure O_2 at ambient pressure. In a typical test, BnOH (0.8 mL), o-xylene (0.8 mL) and the given particle at constant weight loading (1 wt% respect to the liquid mixture), were added to a Schlenk (50 mL) with a magnetic stirring bar. The Schlenk tube was connected with an O_2 gasbag. In the latter case, the Schlenk was connected to a condenser with a silicon rubber cap on top. The gas environment in the Schlenk was purged three times with pure O_2 for 5 s. In a typical test, the Schlenk was stirred at 100 °C for 60 min at 1500 rpm. After the reaction, acetone was added to destabilize the as-generated foam. Then, the liquid was centrifuged (7000 rpm) for 2 min and the supernatant solution was recovered using a syringe.

The solution was analyzed using an Agilent 7820A GC equipped with a flame ionization detector (FID) detector and an HP-5 column (length 30 m, i.d. 0.25 mm). Mass balance errors were within 5% for all catalytic tests. The BnOH conversion and BnAH yield were calculated by interpolation of the corresponding calibration curves using biphenyl as internal standard as follows

$$\text{BnOH conversion}(t) = 1 - \frac{n_{BnOH}(t)}{n^0_{BnOH}} x100 \qquad (S1)$$

$$\text{BnAH yield}(t) = \frac{n_{BnHA}(t)}{n^0_{BnOH}} x100 \qquad (S2)$$

where n^0_{BnOH} and $n^0_{BnOH}(t)$ refer to the mole number of BnOH at time = 0 and time = t, respectively, and $n_{BnAH}(t)$ is the mole number of benzaldehyde at time = t.

The turnover number (TON) with respect to benzaldehyde formation were computed as follows

$$TON(t) = \frac{n_{BnHA}(t)}{n_{Pd}\, D_{Pd}} \qquad (S3)$$

Catalyst reusability test

A reusability test was performed to evaluate the stability of Pd/JPs. After each catalytic run, the particles were separated from the reaction media by centrifugation (7000 rpm for 3 min), washed with acetone four times, and dried at 80 °C for at least 4 h before reuse in the next run.

Foam stability tests

The BnOH/o-xylene foams were prepared as follows. First, the given particles were placed in a 50-mL Schlenk with BnOH (0.8 mL) and o-xylene (0.8 mL) and ultrasonicated at room temperature for 30 min until optimal wetting. Subsequently, the dispersion was stirred in a silicon oil bath at 100 °C for 60 min with 1500 rpm stirring rate. After preparation, the foam was kept static at room temperature to evaluate its stability. The foam height was measured by direct inspection of the foam using a ruler. A Leica DM750 optical microscope with GXCAM software, 10× ocular, 4×, 10×, 40 x, and 100× objectives was used to measure the bubble size. ImageJ software was used to quantify the average droplet size.

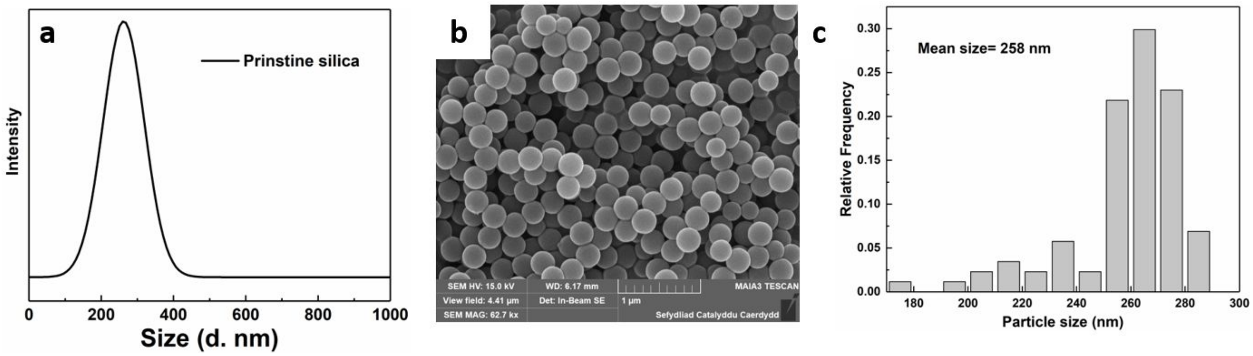

Figure S1. (a) Dynamic light scattering (DLS) result for pristine silica particles. (b) Scanning electron microscope (SEM) image of pristine silica particles. (c) Particle size distribution of Pristine silica.

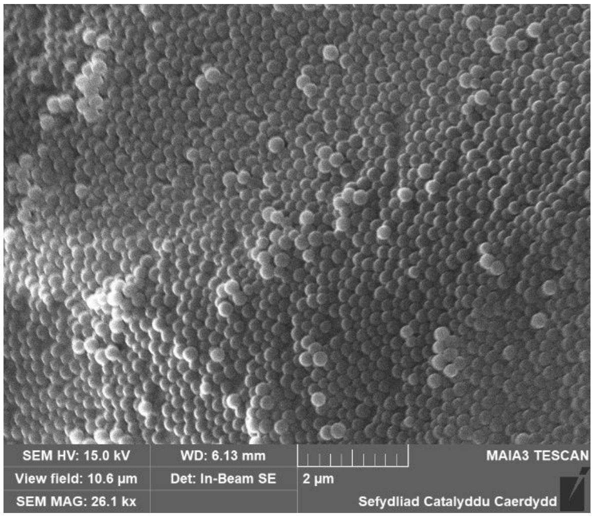

Figure S2. SEM image of the Stöber silica particles embeds in the wax droplet in half.

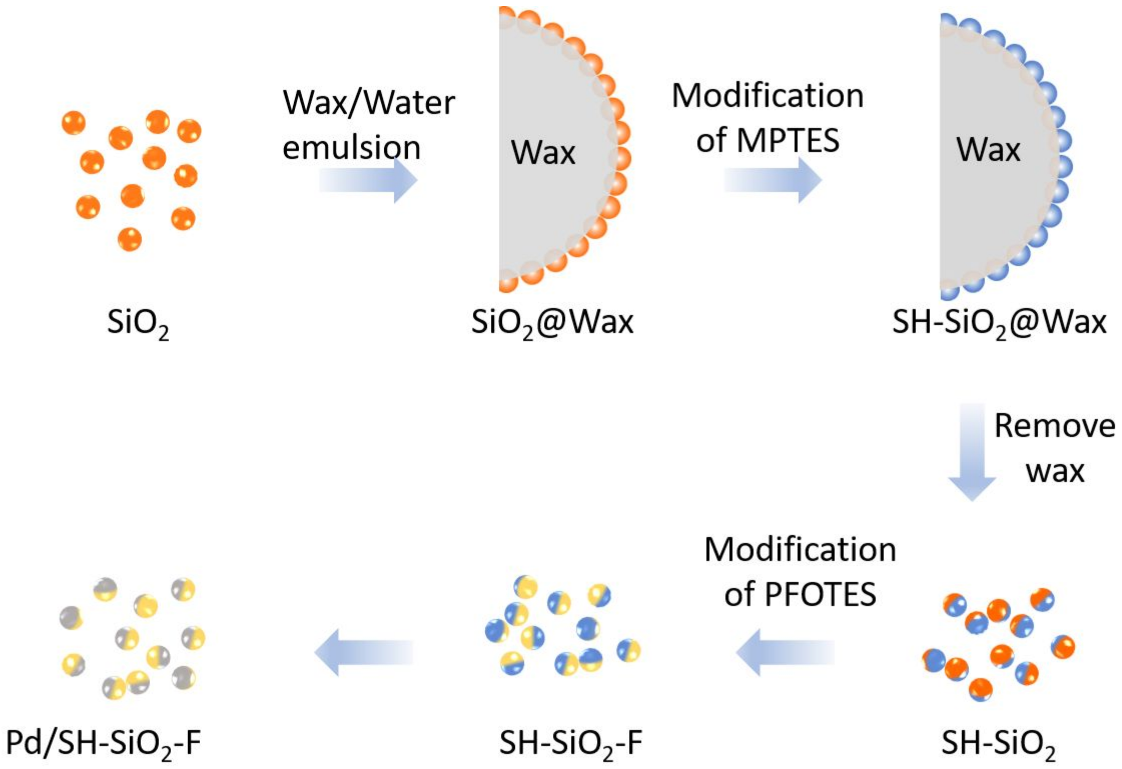

Figure S3. General scheme of the synthesis protocol of JPs.

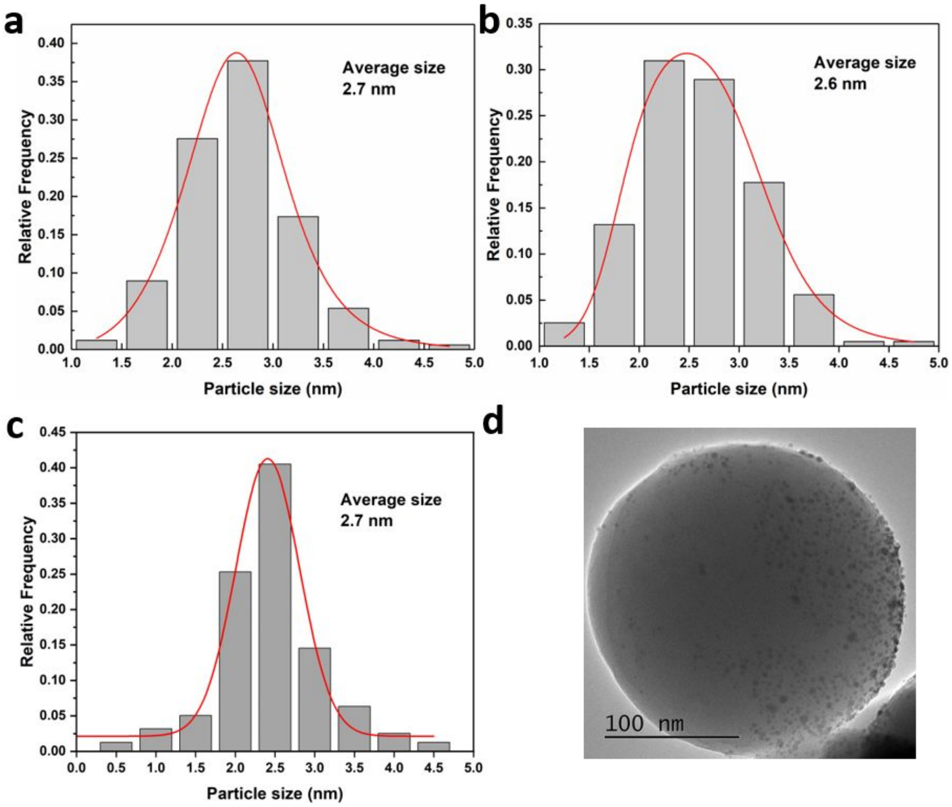

Figure S4. Size distribution of Pd nanoparticles in (a) fresh Pd/Non-JPs, (b) fresh Pd/JPs, (c) spent Pd/JPs after five consecutive catalytic runs, and (d) TEM image of spent Pd/JPs after five consecutive catalytic runs.

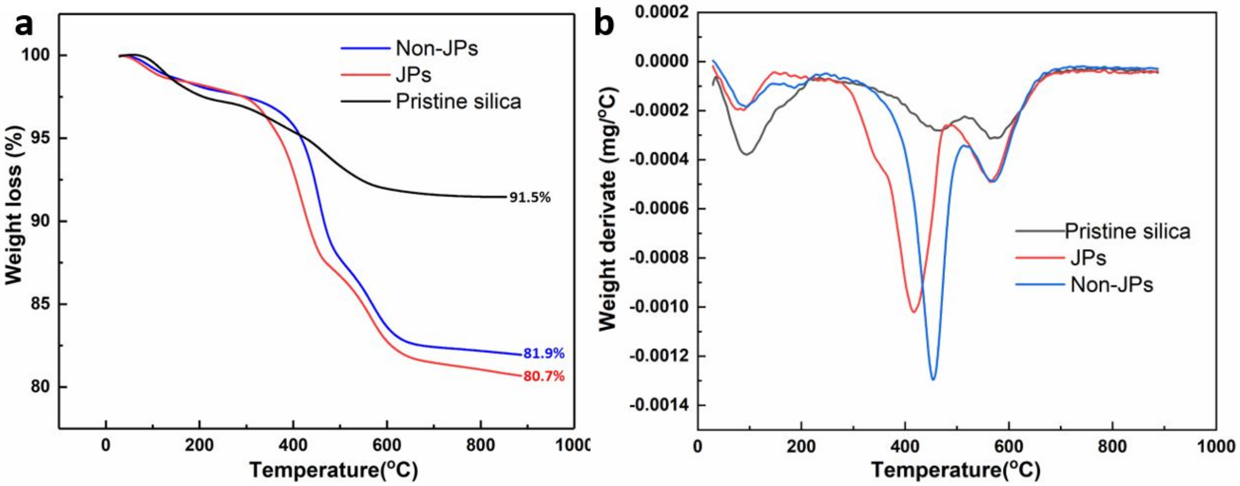

Figure S5. (a) TG and (b) DTG profiles of pristine silica, JPs and Non-JPs in air atmosphere.

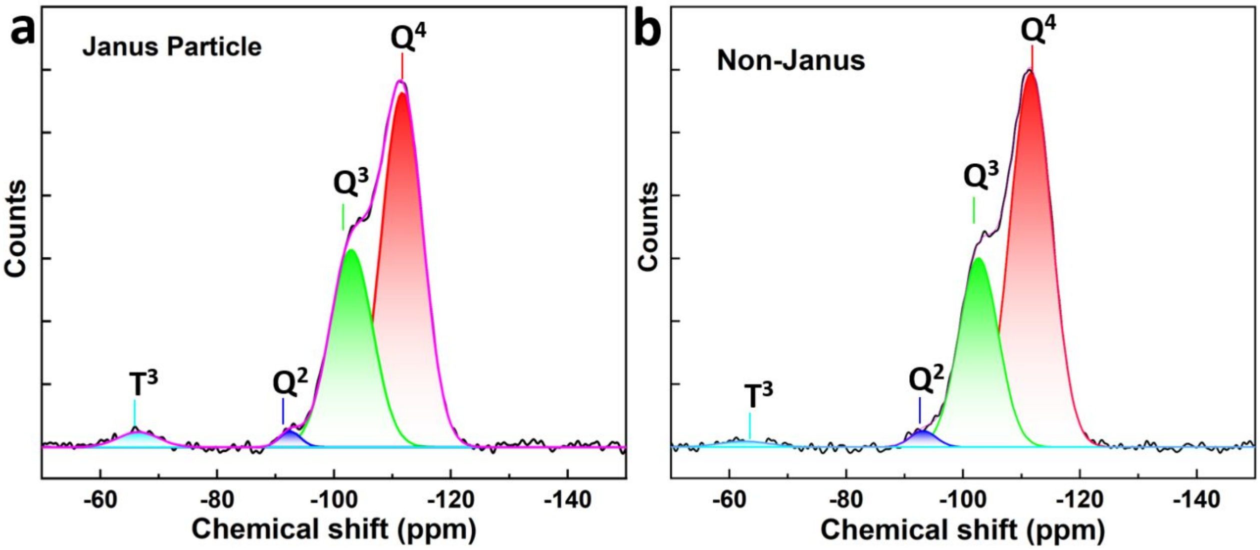

Figure S6. Solid-state ^{29}Si NMR MAS spectra of **(a)** JPs and **(b)** Non-JPs.

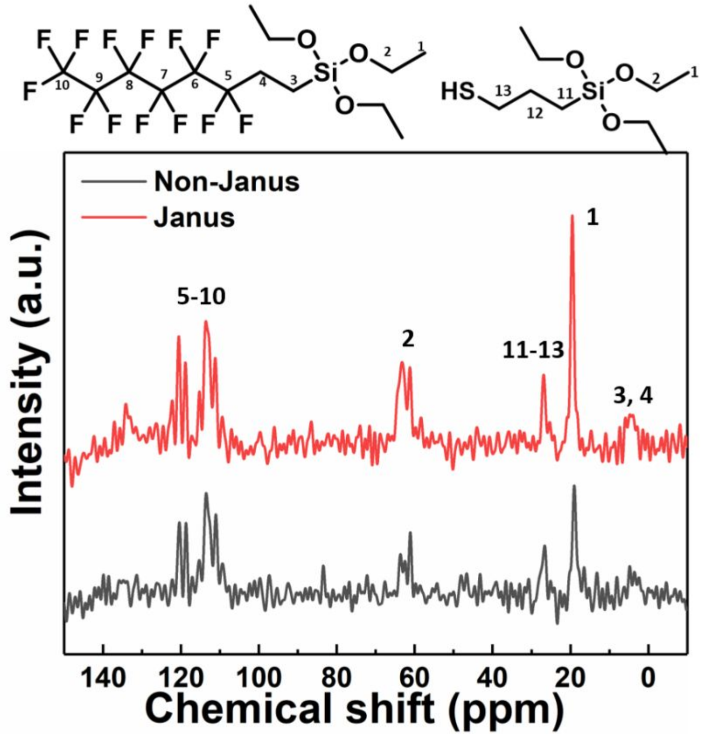

Figure S7. Solid-state ^{13}C NMR MAS spectra of JPs and Non-JPs.

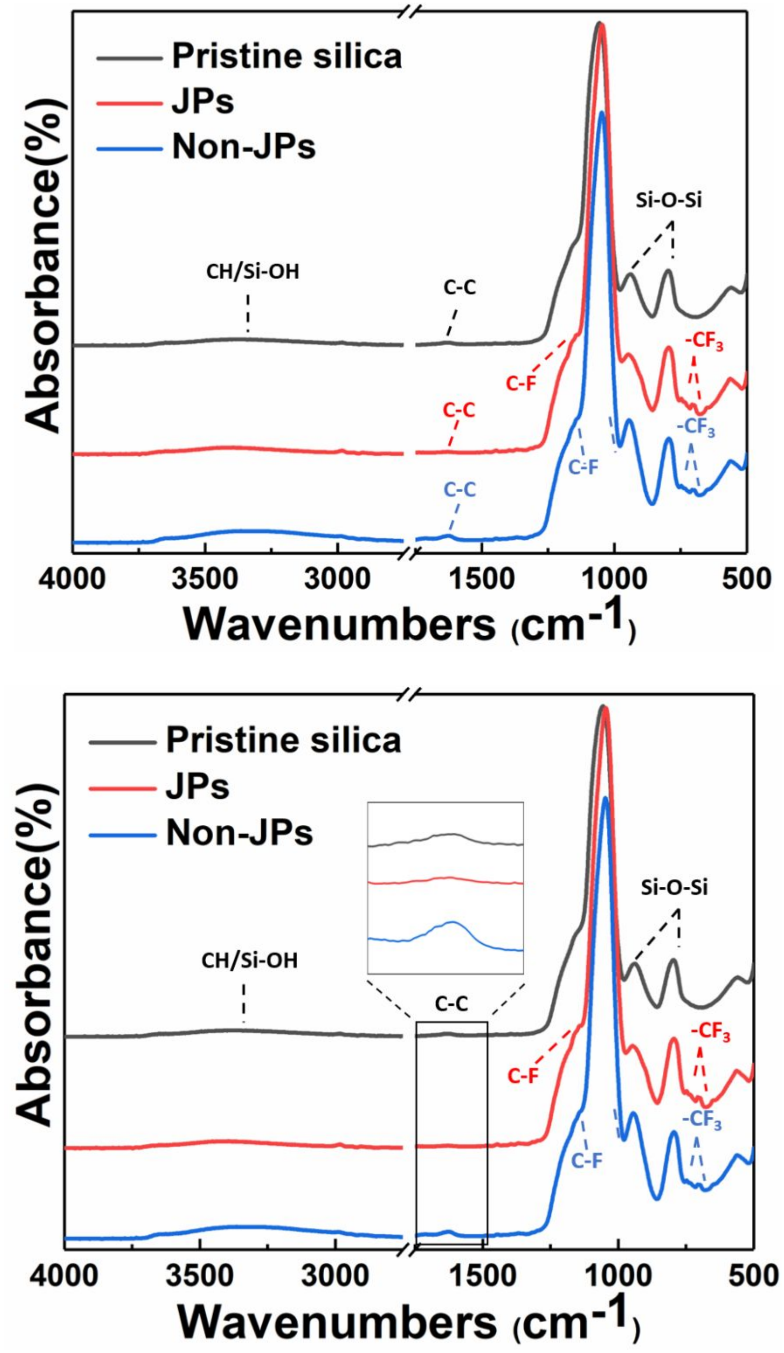

Figure S8. FT-IR spectroscopy of pristine silica, JPs and Non-JPs.

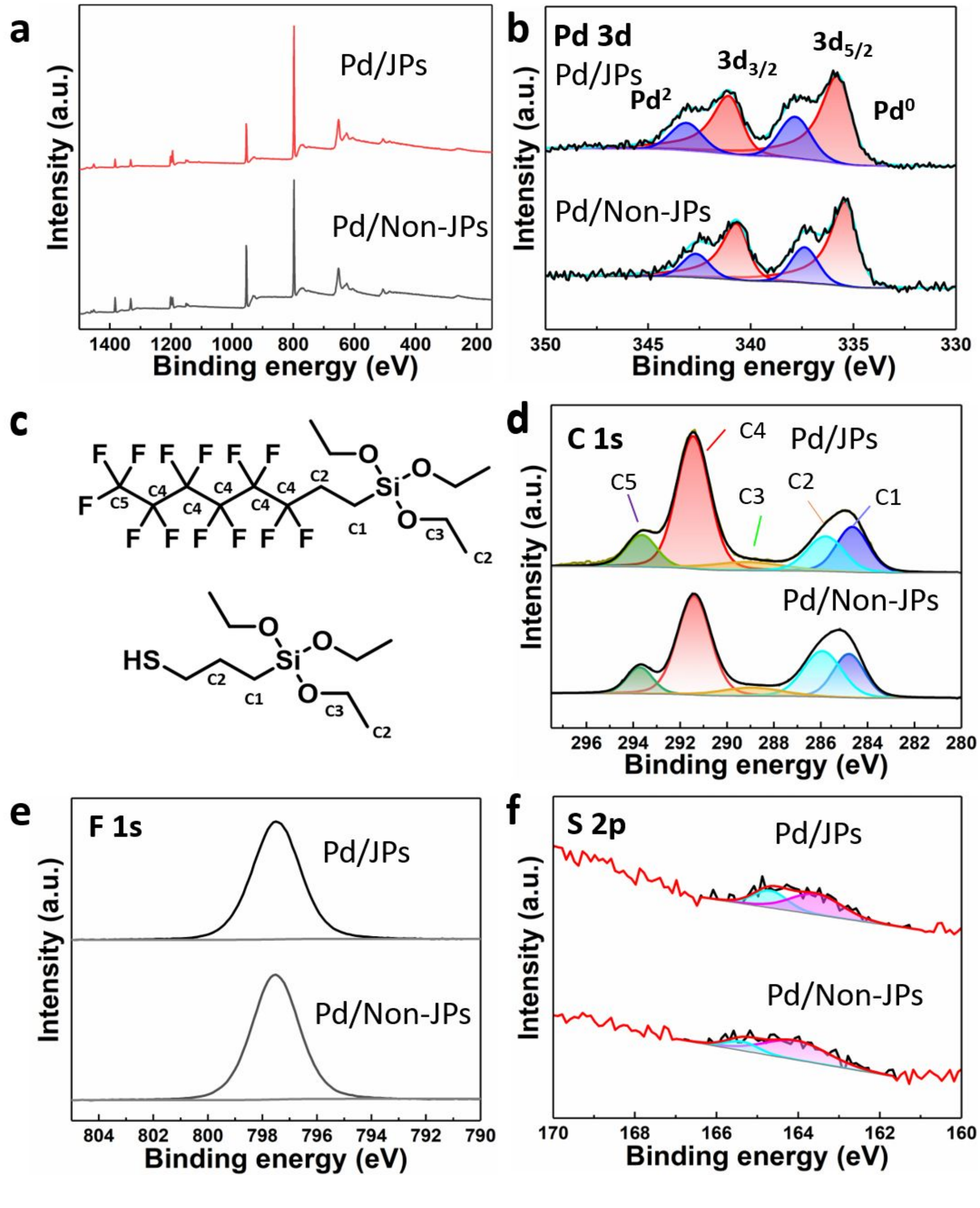

Figure S9. (a) X-ray photoelectron spectroscopy (XPS) survey spectrum of Pd/JPs and Pd/Non-JPs surface. (b) High-resolution narrow scan of the two particles for Pd(3d). (c) Molecular structure of the silanes grafted on the surface of silica particles. (d) High-resolution deconvoluted spectra of the two particles for C(1s). (e) High-resolution narrow scan of the two particles for F(1s). (f) High-resolution narrow scan of the two particles for S(2p).

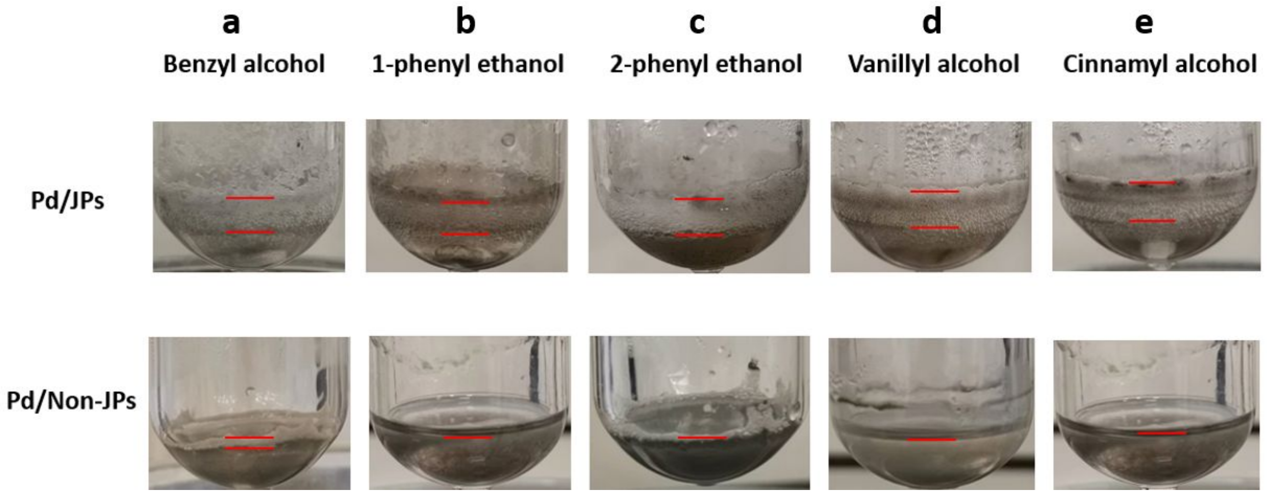

Figure S10. Foamability of Pd/JPs and Pd/Non-JPs in different solvents; (a) Benzyl alcohol, (b) 1-phenyl ethanol, (c) 2-phenyl ethanol, (d) Vanillyl alcohol, (e) Cinnamyl alcohol. Conditions: 0.8 mL of aromatic alcohol, 0.8 mL of o-xylene or dodecane, 1 wt % particle, 1 bar O_2, 1500 rpm, 100-120 °C, 1 h.

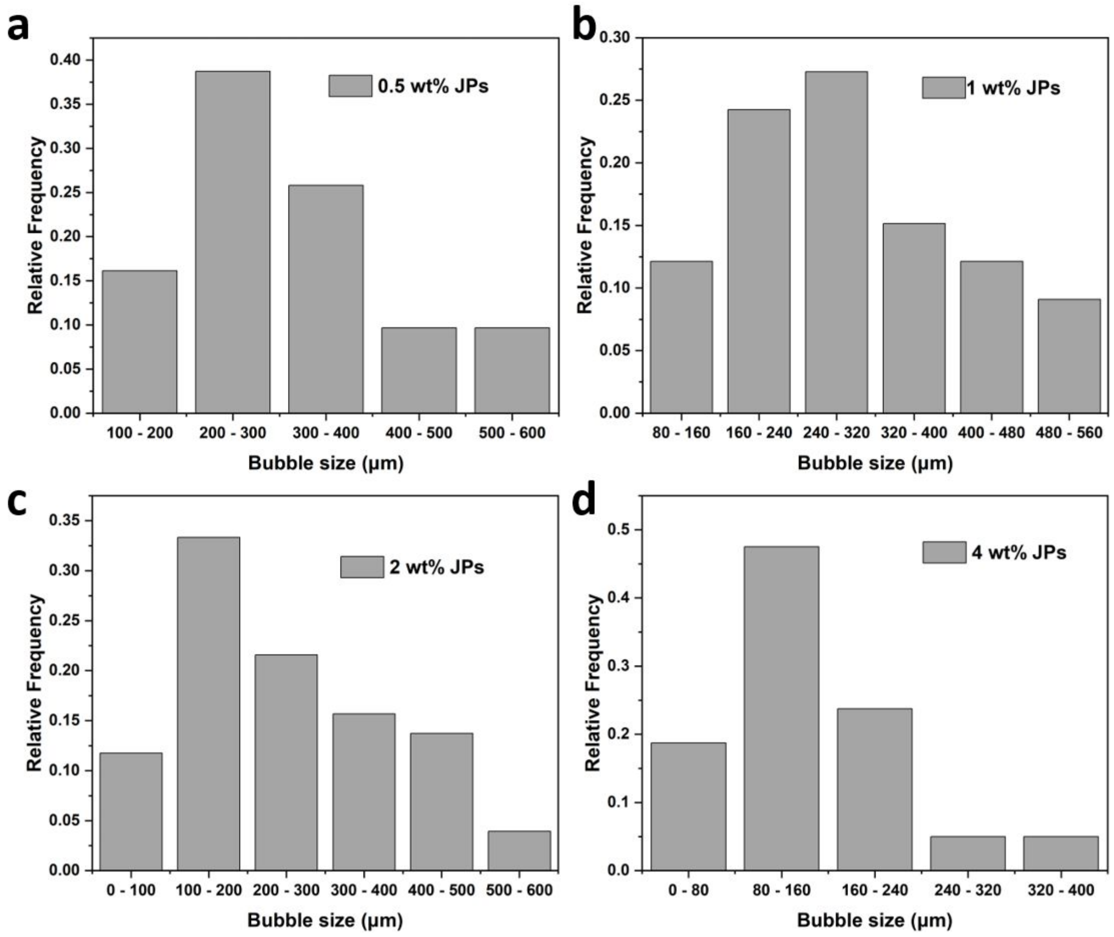

Figure S11. Bubble size distribution for foams prepared with JPs at variable weight loading. Foaming conditions: 0.8 mL of BnOH, 0.8 mL of o-xylene, 0.5-4.0wt% JPs, 1500 rpm, 100 °C, 1 h.

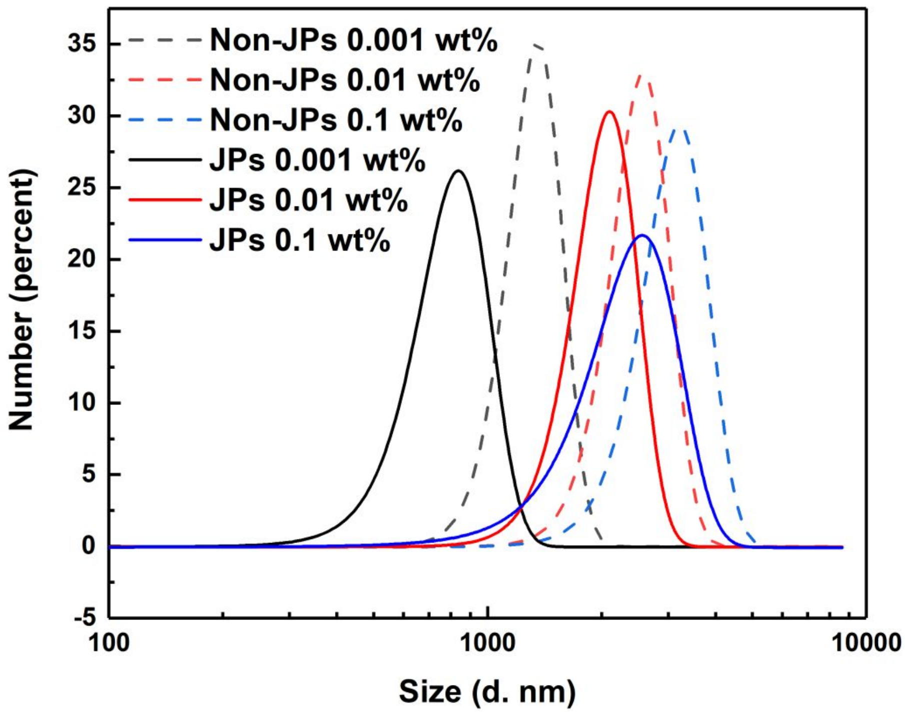

Figure S12. Size distributions for JPs and Non-JPs at 0.001 wt%, 0.01 wt%, and 0.1 wt% in BnOH/o-xylene mixture (1:1 v/v) measured by DLS.

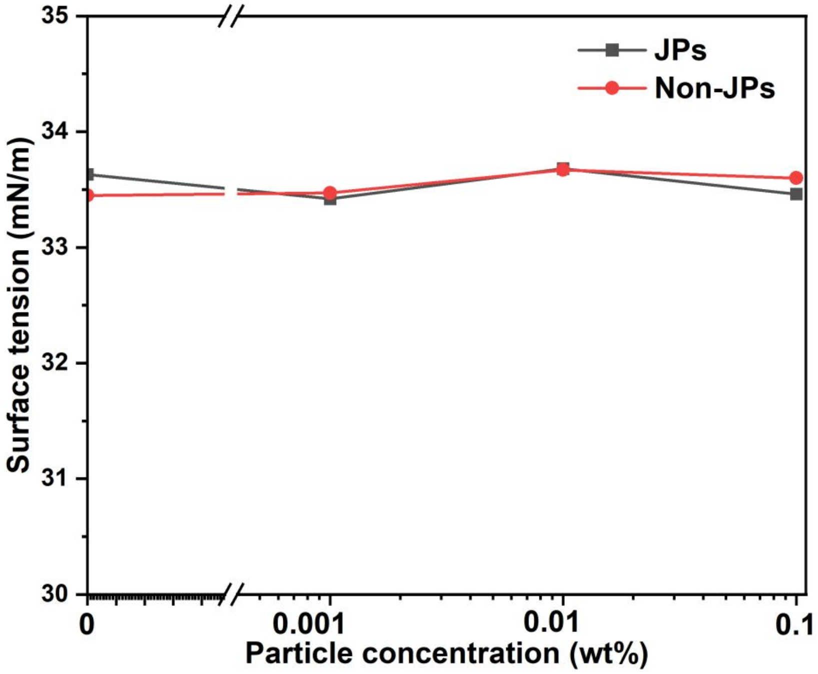

Figure S13. Surface tension of BnOH/o-xylene mixture (1:1 v/v) with the concentration of 0.001 wt%, 0.01 wt%, and 0.1 wt% JPs and Non-JPs.

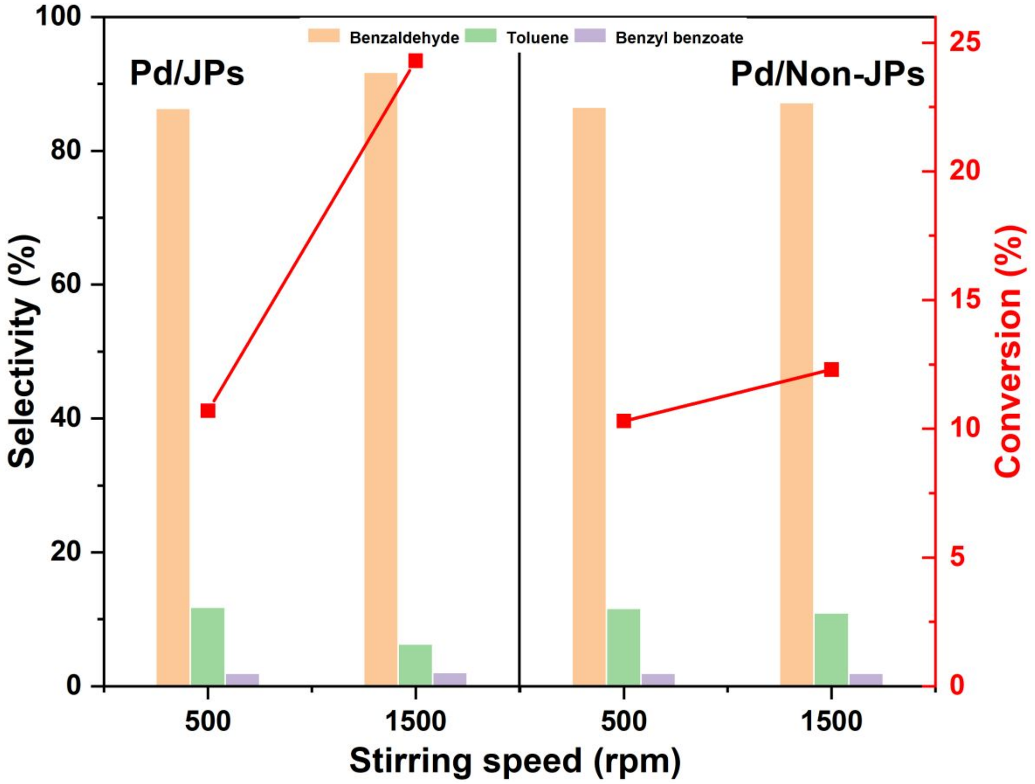

Figure S14. BnOH conversion and selectivity to different products in the aerobic oxidation of BnOH over Pd/JPs and Pd/ Non-JPs with and without foam, respectively. Reaction conditions: 0.8 mL of BnOH, 0.8 mL of o-xylene, 1wt% catalysts, 500-1500 rpm, 100 °C, 1 h.

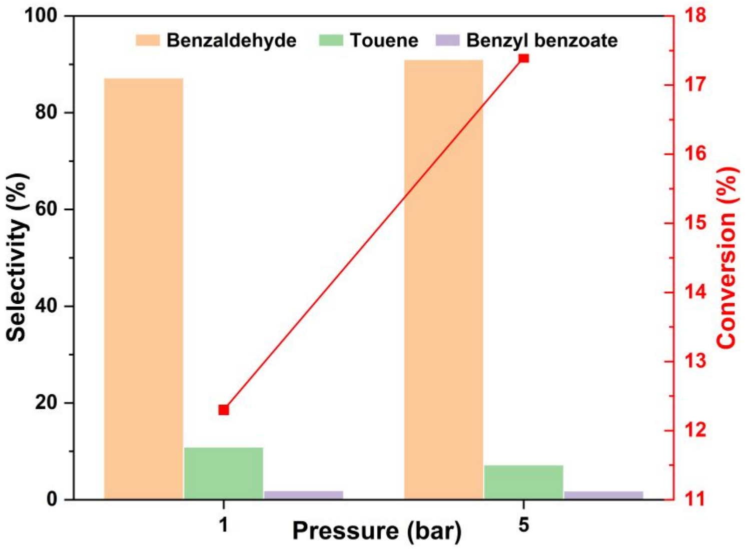

Figure S15. BnOH conversion and selectivity to different products in the aerobic oxidation of BnOH over Pd/Non-JPs with different 1 and 5 bar O_2 pressure. Reaction conditions: 0.8 mL of BnOH, 0.8 mL of o-xylene, 1wt% Pd/Non-JPs, 1500 rpm, 100 °C, 1 h.

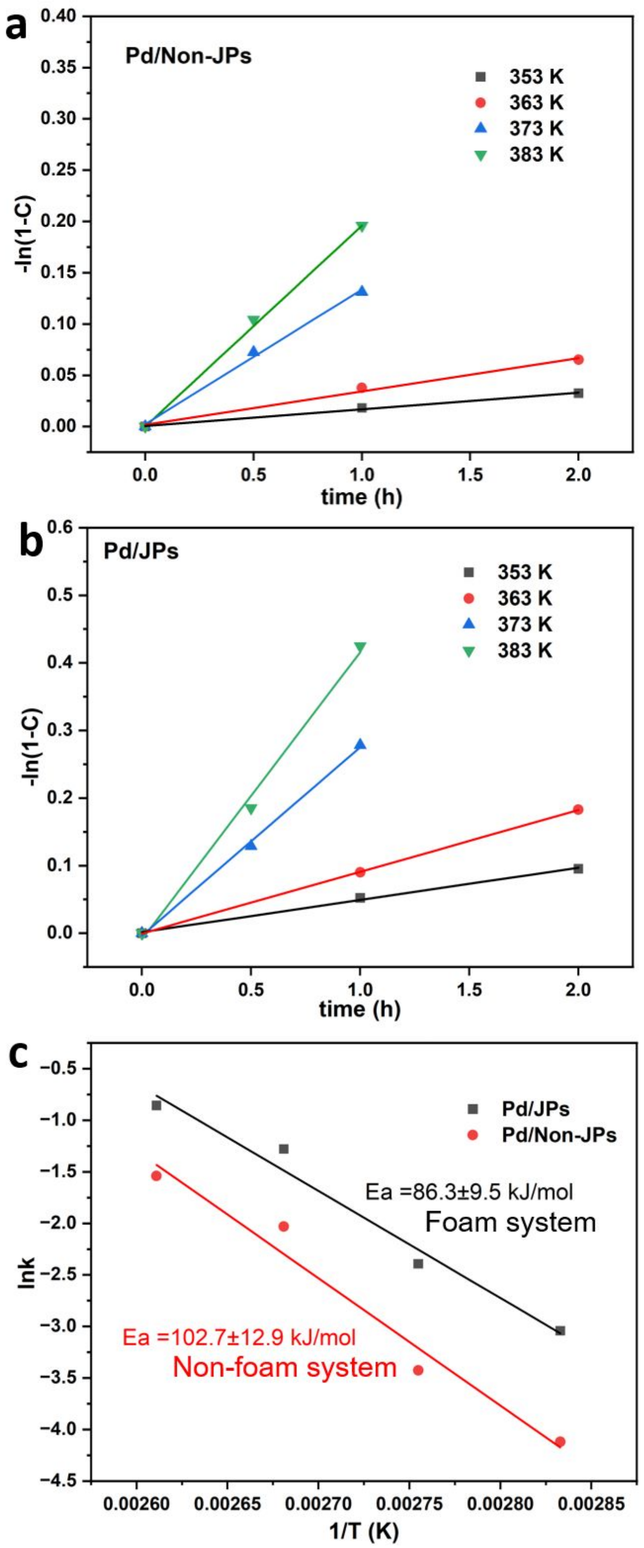

Figure S16. Kinetic plots of –Ln(1-C) against time for the aerobic oxidation of BnOH catalyzed by (a) Pd/Non-JPs (without foam) and (b) Pd/JPs (with foam); (c) Variation of TOF at time t=0 for with reciprocal temperature for Pd/JPs and non-JPs.

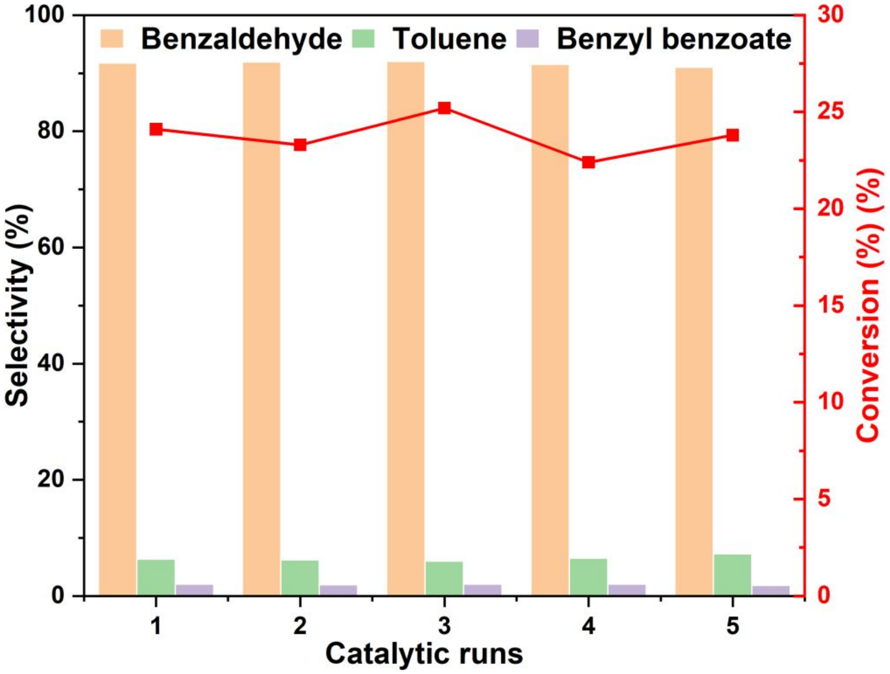

Figure S17. BnOH conversion and selectivity to the different products in the aerobic oxidation of BnOH over Pd/JPs in foam in five consecutive runs. Reaction conditions: 0.8 mL of BnOH, 0.8 mL of o-xylene, 1wt% Pd/JPs, 1500 rpm, 100 °C, 1 h.

Table S1. Pd concentration in the catalysts before and after reaction (measured by ICP-MS)

Catalyst	Before reaction (mg/g)	After reaction (mg/g)	After 5th Run (mg/g)
Pd/JPs	8.14 ± 0.020	8.13 ± 0.013	8.13 ± 0.021
Pd/Non-JPs	8.21 ± 0.024	8.22 ± 0.017	-

- not studied

References

[S1] Borodzinski, A.; Bonarowska, M. Relation between Crystallite Size and Dispersion on Supported Metal Catalysts, *Langmuir* **1997**, 13, 5613-5620.
